# Computational Identification of Transcriptional Regulators in Human Endotoxemia

**DOI:** 10.1371/journal.pone.0018889

**Published:** 2011-05-27

**Authors:** Tung T. Nguyen, Panagiota T. Foteinou, Steven E. Calvano, Stephen F. Lowry, Ioannis P. Androulakis

**Affiliations:** 1 BioMaPS Institute for Quantitative Biology, Rutgers University, Piscataway, New Jersey, United States of America; 2 Department of Biomedical Engineering, Rutgers University, Piscataway, New Jersey, United States of America; 3 Department of Surgery, Robert Wood Johnson Medical School, University of Medicine and Dentistry, New Jersey, New Brunswick, New Jersey, United States of America; Kyushu Institute of Technology, Japan

## Abstract

One of the great challenges in the post-genomic era is to decipher the underlying principles governing the dynamics of biological responses. As modulating gene expression levels is among the key regulatory responses of an organism to changes in its environment, identifying biologically relevant transcriptional regulators and their putative regulatory interactions with target genes is an essential step towards studying the complex dynamics of transcriptional regulation. We present an analysis that integrates various computational and biological aspects to explore the transcriptional regulation of systemic inflammatory responses through a human endotoxemia model. Given a high-dimensional transcriptional profiling dataset from human blood leukocytes, an elementary set of temporal dynamic responses which capture the essence of a pro-inflammatory phase, a counter-regulatory response and a dysregulation in leukocyte bioenergetics has been extracted. Upon identification of these expression patterns, fourteen inflammation-specific gene batteries that represent groups of hypothetically ‘coregulated’ genes are proposed. Subsequently, statistically significant cis-regulatory modules (CRMs) are identified and decomposed into a list of critical transcription factors (34) that are validated largely on primary literature. Finally, our analysis further allows for the construction of a dynamic representation of the temporal transcriptional regulatory program across the host, deciphering possible combinatorial interactions among factors under which they might be active. Although much remains to be explored, this study has computationally identified key transcription factors and proposed a putative time-dependent transcriptional regulatory program associated with critical transcriptional inflammatory responses. These results provide a solid foundation for future investigations to elucidate the underlying transcriptional regulatory mechanisms under the host inflammatory response. Also, the assumption that coexpressed genes that are functionally relevant are more likely to share some common transcriptional regulatory mechanism seems to be promising, making the proposed framework become essential in unravelling context-specific transcriptional regulatory interactions underlying diverse mammalian biological processes.

## Introduction

Inflammation and activation of innate immunity are essential defense responses against invading pathogens and endogenous danger signals. The innate immune response involves the initial recognition of conserved pathogen-associated molecular patterns by members of the Toll-like receptor (TLR) family [1]. The exposure of the host to gram negative bacteria, simulated by lipopolysaccharide (LPS) recognized by TLR-4, triggers intracellular signalling cascades which eventually release a lot of pro- and anti- inflammatory cytokines [2]. While the host inflammatory response is essential to resolve the infection or repair the damage and restore the system homeostasis, it also plays a central pathogenic role in a wide spectrum of diseases including sepsis [3]. Under healthy circumstances, inflammatory responses are activated, clear the pathogen in the case of infection, initialize a repair process and then abate [4]. However when anti-inflammatory processes fail, an amplified inflammation can turn what is normally a beneficial reparative process into a detrimental physiological state with severe, uncontrolled systemic inflammation [5].

Studies involving experimental human endotoxemia have reported rapid intravenous infusion in doses of 2–4 ng/kg body weight, which effectively induces an acute systemic inflammatory condition that mimics the early flow phase of injury and infection [6], [7], [8], [9], [10]. In human peripheral blood leukocytes, intravenous administration of endotoxin elicits dynamic and reproducible changes in the circulating leukocyte population as well as significant changes in blood leukocyte gene expression patterns [11]. This perturbation of leukocyte gene expression involves several thousands of transcripts and accompanies the systemic physiological responses during inflammation, which peaks ∼4–6 hours after endotoxin exposure and resolves within 24 hours, compatible with a large and dynamic regulatory network.

Transcriptional regulation is driven by incoming signals which activate transcription factors (TFs) through mechanisms such as phosphorylation or dimerization. Activated complexes are subsequently translocated into the nucleus and bind to the promoter region of target genes in the genome in order to activate or repress gene expression [12]. It is hypothesized that this regulation process is mainly controlled by the interplay between TFs and their corresponding transcription factor binding sites (TFBSs) on the proximal promoters of the target genes [13], [14], [15]. Recent technological developments have enabled the ability to broadly assess TF activities at a genome-wide scale. However, there is still no transcription factor-focused method that enables monitoring of all TFs at a time; technologies such as Chip-on-chip [16], [17], SELEX [18], [19] can identify all DNA binding sites occupied by a single TF given a condition. In order to compensate for this inability, computational techniques have become an essential tool in predicting putative TFBSs at a large scale [20], [21].

Due to the fact that TFs in higher organisms regulate gene expression in a combinatorial manner rather than in isolation [22], [23] and that TFBSs tend to form clusters of binding sites, known as *cis*-regulatory modules (CRMs) [24], [25], computational methods have shifted towards discovering CRMs instead of a single TFBS. A *cis*-regulatory module is generally considered as the smallest functional regulatory unit [26]. From a computational standpoint, such module is mainly characterized by two factors: (i) composition which consists of a set of non-overlapping binding sites of TFs on the control regions of a gene and (ii) structural constraints that take into account the strand orientation to which TFs bind, the order and the distance between successive binding sites [27]. A variety of methods have been proposed to search for CRMs that include the structural constraints (FrameWorker [28], CMA [29]) or without the structural constraints (CREME [30], ModuleMiner [31], Stubb [32]). Some methods attempt to incorporate *a priori* knowledge of CRMs (HexDiff [33], ESPERR [34], [35]) to increase the specificity of the prediction while some others are purely computationally discovery of CRMs (CisModule [36], CSam and D2Z-set [37]).

However, given a methodology to search for CRMs, a critical issue in predicting functional binding sites is identifying a set of relevant promoters that share common *cis*-regulatory modules or alternatively a set of genes that are potentially coregulated [38]. As such it is more appropriate to explore the concept of ‘gene battery’ originally proposed by Britten and Davidson [39] and has been further explored in the literature [40], [41], [42], [43]. A gene battery refers to a group of genes that are coordinately expressed and/or functionally coupled since their regulatory regions respond to the same transcriptional signals [37], [44]. With the assumption that genes in a gene battery are involved in key biological processes, recognized CRMs will consist of putative functional binding sites that are associated with essential transcriptional regulators. Yet, in higher eukaryotes especially in humans the problem turns to be much more difficult. One of the most critical issues is to determine which genes belong to the same gene battery. Prior studies assume that either coexpressed genes [45], [46], [47] or genes that belong to the same biological process [48], [49] could be governed by some common regulatory mechanism. However, recent evidence suggests that co-expression or co-function alone is not sufficient to infer the existence of common regulatory mechanisms [50], [51]. Oftentimes co-expressed genes can participate in a diverse array of biological functions while functionally-relevant genes can be characterized by different expression patterns [52], [53]. Predicated upon these, in this study we explore the possibility that genes that are both co-expressed and functionally-relevant may be more likely to be co-regulated. Since genes within the same pathway encode for a set of interacting proteins, they are more likely to be governed by some common regulatory mechanism [54]. Therefore, the unifying hypothesis of this study is that genes that participate in the same pathway are functional relevant.

In this study, given the transcriptional profiling analysis of human blood leukocytes we hypothesized that genes that are most responsive to an external perturbation (endotoxin) and have concerted changes in their expression profiles are governed by some common regulatory mechanism. Based on our prior work, high-dimensional microarray data are decomposed into a comprehensive set of temporal responses that, in the case of transient human endotoxemia, capture the essence of a pro-inflammatory phase, a counter-regulatory response as well as a dysregulation in leukocyte bioenergetics [55]. Upon identification of these patterns, a number of inflammation-specific pathways are selected by evaluating the enrichment of the corresponding subsets and thereby defining a putative set of ‘coregulated’ genes. The CRM-se arching process, similar to FrameWorker [28], is proposed with a novel heuristic to address the issue related to multiple alternative promoters in eukaryotic genes. The definition of CRM structural constraints has been adjusted so that no parameter is required for the searching process except for the statistically significant threshold for the CRM selection. Furthermore, motivated by the work of Schones et al. [56] a pre-compilation step is performed by converting all promoter sequences into a set of corresponding promoter profiles of binding sites improving the estimation of the statistical significance of recognized CRMs. Overall, the present study aims to computationally identify transcription factors that are crucial to the dynamics of essential physiological processes associated with the acute human inflammatory response and provides significant insights into putative transcriptional regulatory mechanisms that underlie gene expression. Computational results were verified primarily based on available literature.

## Results

### Identification of putatively ‘coregulated’ genes

**Figure 1:**
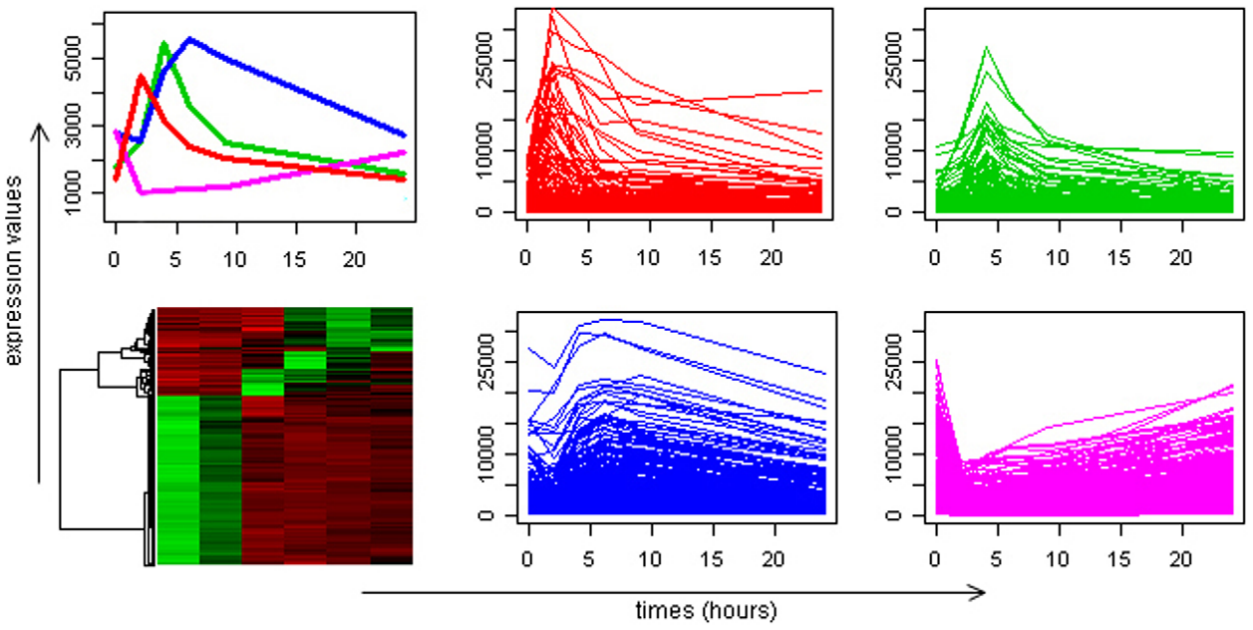
Critical responses to human inflammation. Gene expression patterns selected from the LPS dataset, including early up – 182 probesets (red), middle up – 119 probesets (green), late up – 284 probesets (blue), and down – 1,118 probesets (magenta); totally 1,730 selected probesets over 3,269. Top-left is the average expression profiles of these patterns; bottom-left is the corresponding heat-map; and the rest are expression profiles of selected genes in four patterns (the horizontal axis is six time-points (0, 2, 4, 6, 9, 24 hours) and the vertical axis is the intensity of mRNA levels).

Given the assumption that genes that are co-expressed and functionally relevant are more likely to be co-regulated, we first identify significant expression patterns from *in vivo* human transcriptional data [11]. Based on 3,269 differentially expressed probesets, we explored the potential of our prior work to identify highly coexpressed genes [55]. Specifically, the algorithm performs a consensus clustering and a trivial cluster removal procedure resulting in four expression patterns that describe the dynamic evolution of endotoxin-induced human inflammation (**Figure 1**). The ‘early-up’ pattern consists of genes that are involved in critical pro-inflammatory processes (e.g. TNF, NFkBIA, C-X-C motifs – CXCL1, CXCL2, and CCL20). The ‘middle-up’ pattern represents an increased expression of genes with the peak at 4 hrs post-endotoxin administration, containing inflammatory-relevant signaling pathways such as Apoptosis, Toll like receptor (TLR) signaling. The ‘late-up’ pattern characterizes anti-inflammatory processes and the ‘down’ pattern which is the most populated expression motif is characterized by genes involved in cellular bio-energetic processes e.g. oxidative phosphorylation, ribosome biogenesis and assembly.

Since pathways are robust and flexible enough to ensure cell survival under environmental changes, the corresponding genes are more likely to be characterized by a stronger coherency rather than those in a gene ontology (GO) definition. Based on the hypothesis that genes encoding a set of related proteins are more likely to be transcribed under some common regulatory mechanisms [54], we opt to use biological information in the context of pathways (KEGG database) rather than GO terms. All genes that participate in the same pathway are considered to be functionally relevant are related [53]. In each expression pattern, we select statistically inflammatory relevant significant pathways (p-value<0.05) based on literature information [55]. Accordingly, fourteen sets of genes that belong to a specific pathway and a pattern of gene expression are extracted. We further assume that these groups of genes represent gene batteries, and hereby are more likely to be coregulated (**Table 1**).

**Table 1:** Data information and inflammation-relevant significant functions.

Expression data (3,269 probesets*)			Relevant significant functions (p-value<0.05)	
Patterns	# of probesets(Total: 1703)	# of genes+(Total: 1213)	Pathways (KEGG)	Corresponding selected genes
**Early-up**	182	141	Apoptosis^1^	il1a, il1b, nfkbia, tnf
			Cytokine-cytokine receptor interaction^1^	ccl20,ccl4, cxcl1, cxcl2, il1a, il1b, il8, inhbb, tnf
			Toll-like receptor signaling pathway^1^	ccl4, il1b,il8, map2k6, nfkbia, tnf
**Middle-up**	119	88	Apoptosis^1^	casp10, cflar, fas, irak3, myd88, nfkb1, nfkb2, rela
			Toll-like receptor signaling pathway^1^	myd88, nfkb1, nfkb2, rela
**Late-up**	284	185	Apoptosis^1^	casp8, il1r1, il1rap, irak4, pik3cg, tnfrsf10c, tnfsf10
			Cytokine-cytokine receptor interaction^1^	ccr1, csf3r, il10rb, il13ra1, il1r1, il1rap, il8ra, il8rb, tnfrsf10c, tnfsf10
			Toll-like receptor signaling pathway^1^	casp8, irak4, pik3cg, tlr1, tlr5, tlr8
			Jak-STAT signaling pathway^1^	csf3r, il10rb, il13ra1, pik3cg, stat2, stat5b
**Down**	1118	799	Citrate cycle (TCA cycle)^2^	acly, idh2, idh3a, mdh1, mdh2, suclg2
			Pyrimidine metabolism^2^	dck, dctd, dut, entpd6, pole3, polr2b, polr2e, polr2k, rpa1, uckl1
			Pyruvate metabolism^2^	akr1b1, glo1, ldhb, mdh1, mdh2, pdhb
			Ribosome^1^	fau, rpl10a, rpl12, rpl13a, rpl14, rpl18, rpl24, rpl27, rpl27a, rpl29, rpl3, rpl36a, rpl36al, rpl37a, rpl38, rpl8, rps2, rps24, rps7, rps9
			Oxidative phosphorylation^2^	atp5a1, atp5b, atp5f1, atp5g1, atp5g2, atp5g3, atp5i, atp5h, atp5j2, atp5l, atp5o, atp61f, cox4i1, cox5a, cox6c, cox7c, cyc1, nduf1, ndufa13, ndufa3,ndufa4, ndufa5, ndufa6, ndufab1, ndufb2, ndufb4, ndufb5, ndufb8, ndufc2, ndufs4, ndufs5, ndufs6, ndufs7, ndufs8, ppa2, ucrc, uqcrb, uqcrc2, uqcrh, uqcrq

*: 3,269 significantly differentially expressed probesets were selected by ANOVA (p-value<10^−4^) from the total 44,924 probesets;

+ : the number of corresponding genes with promoter annotation in Genomatix;

1 : regulatory pathways;

2 : metabolic pathways.

### Statistical significance of common CRMs

**Figure 2:**
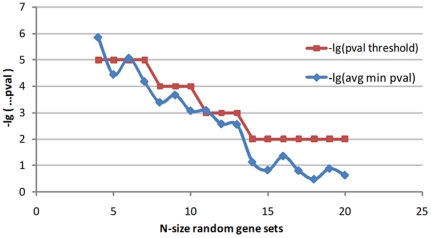
Statistical significance thresholds of CRMs. A procedure randomly picks a gene-set with N genes from the background and search for common CRMs (δ = 0.7) in that gene-set. The statistical significant p-value for each CRM is estimated and the minimum one is reported. Each point in the blue curve is a transformed value of the mean of the minimum p-values of CRMs in 100 times running the procedure for the corresponding k. Approximately, the red curve shows which thresholds should be used for the non-random cases. After N = 14 genes, only one threshold is used to ensure the significance (p-value = 0.01).

Within a gene battery, CRMs that are present on the control regions of corresponding genes above a frequency threshold (e.g. δ = 70% of the number of genes) are considered as common CRMs. However, such CRMs can also be overrepresented in random gene sets. Therefore, in order to restrict the false positive matches and increase the statistical power of our method, we estimate the hyper-geometric p-values of common CRMs vs. a background set and only select those CRMs whose p-values exceed a pre-defined statistically significance threshold (e.g. 10^−4^). However, this threshold is very sensitive to the size of the gene battery and thus a uniform significance threshold cannot be applied for all gene batteries. As a result, we developed a heuristic procedure for estimating the significance threshold of common CRMs with respect to the size of gene batteries. The procedure is repeated 100 times for each N-size gene-set (N = 4, 5…, 20). At each iteration, the algorithm randomly selects N genes from the background set, searches for common CRMs that are present on the promoters of these genes (δ = 0.7), estimates the statistical significance (p-values) for each CRM (see materials and methods), and records the minimum one. In this study, we choose the approximate values of the mean of these minimum p-values to set the criterion for the statistical significance of CRMs in a gene battery with size N (**Figure 2**). Consequently, for each gene battery only those CRMs that are identified with p-values less than the corresponding p-value thresholds are used to infer relevant transcription factors (see Data S1, sheet ‘p-value’).

### Identification of inflammation-relevant transcriptional regulators

One of the key features in our analysis is the identification of significantly overrepresented CRMs in each gene battery (see Materials and methods and Algorithms S1). Based on the size of a gene battery, a corresponding significance threshold is applied to select statistically significant CRMs. Since these recognized CRMs are located on the control regions of many putatively coregulated genes (i.e. a gene battery), they are likely to be composed of functional binding sites that are activated upon the initiation of the transcriptional machinery. We therefore decompose these CRMs into a list of TFBSs to infer associated TFs which can be considered as relevant transcriptional regulators of the corresponding gene battery. In particular, TFs that are present with the high frequency among gene batteries (at least three times across fourteen gene batteries) are assumed to play a key role in the biological process (**Table 2**). We identify 34 transcription factors (TFs) relevant to the human inflammatory responses, of which around 25% has been experimentally shown to be involved in the inflammatory and/or immune response based on literature evidence (discussed below) and more than half of the remaining have been computationally shown to play a critical role in the regulation of immune system [57] (see Data S1, sheet ‘CRMs’, ‘TFs’, and ‘Middle-up TLR’).

**Table 2:** Critical transcription factors in human endotoxemia model.

No.	Patterns	Functions	Transcription factors
1	Early-up	Apoptosis	BRNF, CLOX, E2FF, EKLF, ETSF, HEAT, HOXF, IRFF, MAZF, MYT1, NFKB, RXRF, SORY, SP1F
2	Middle-up	Apoptosis	AP4R, CREB, E2FF, ETSF, GATA, HEAT, MAZF, MZF1, NFKB, NKXH, PAX6, SP1F, ZBPF
3	Late-up	Apoptosis	ATBF, BRNF, CLOX, EBOX, ETSF, FKHD, GATA, HOMF, HOXF, IRFF, NKXH, OCT1, PARF, SORY, STAT, TBPF
4	Early-up	Toll-like receptor signaling pathway	EKLF, HEAT, MAZF, MYT1, SP1F
5	Middle-up	Toll-like receptor signaling pathway	CREB, E2FF, EGRF, EKLF, ETSF, EVI1, HEAT, MAZF, MYBL, MZF1, NFKB, NR2F, PAX6, SORY, SP1F, STAT, ZBPF
6	Late-up	Toll-like receptor signaling pathway	AP4R, ATBF, BRNF, CLOX, ETSF, EVI1, FKHD, GATA, HOMF, HOXF, IRFF, MEF2, NKXH, OCT1, PARF, SORY, STAT, TBPF
7	Early-up	Cytokine-cytokine receptor interaction	SORY, TBPF
8	Late-up	Cytokine-cytokine receptor interaction	AP4R, CLOX, EBOX, ETSF, EVI1, FKHD, GATA, HEAT, HOMF, HOXF, IRFF, MAZF, MEF2, NFKB, NR2F, OCT1, PARF, PAX6, RXRF, SORY, SP1F, TBPF
9	Late-up	Jak-STAT signaling pathway	AP4R, BRNF, CLOX, E2FF, EGRF, ETSF, HEAT, HOMF, HOXF, MAZF, MZF1, RXRF, SP1F, ZBPF
10	Down	Citrate cycle (TCA cycle)	ATBF, BRNF, EGRF, ETSF, FKHD, HEAT, HOMF, HOXF, MAZF, MEF2, MYBL, MYT1, MZF1, NR2F, RXRF, SP1F, STAT, TBPF, ZBPF
11	Down	Pyrimidine metabolism	CREB, E2FF, EBOX, ETSF, IRFF, MYBL, SP1F, ZBPF
12	Down	Pyruvate metabolism	HEAT*
13	Down	Ribosome	E2FF, ETSF, RXRF
14	Down	Oxidative phosphorylation	**None**

*: present in *cis*-regulatory module ‘+HEAT__+NRF1__+NRSF’.

### Putative temporal program of transcriptional regulation

**Figure 3:**
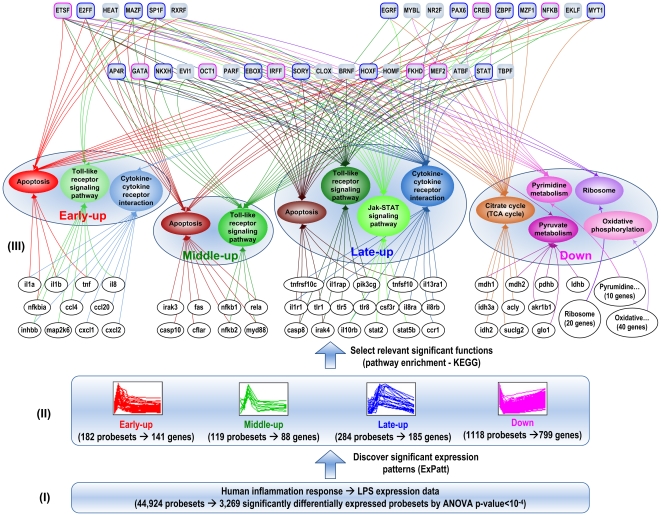
Putative temporal regulatory program in human endotoxemia plus schematic illustration of the integrated computational framework. The clustering and selection step extracts a ‘clusterable’ subset of differentially expressed probesets and cluster it into a number of expression patterns. Subsequently, pathway enrichment is performed in each pattern and relevant significant pathways are selected based on literature information. The process of CRM searching is then applied to each gene battery which is a group of genes that belong to an expression pattern and a particular pathway. Eventually, 34 TFs are identified as human inflammation-relevant transcriptional regulators. The results show a highly dynamic perspective of regulation and interactions between genes, functions, and TF across the time.

The administration of a low dose of endotoxin to human subjects elicits dynamic and reproducible changes in the circulating leukocyte population by altering the expression level of numerous genes. Since the host response to endotoxin evolves dynamically, it is possible to observe a dynamic representation in the transcriptional regulatory program (**Figure 3**). Due to the fact that transcription factors are characterized by pleiotropic effects [58], it is also reasonable to anticipate a significant overlap among sets of transcriptional regulators across various biological processes. On the other hand, our results also illustrate the phenomenon in which genes involved in the same function (pathway) may exhibit different expression patterns and genes within an expression pattern can participate in different functions, implying that there are different regulatory mechanisms regulating genes in the same function or in the same expression pattern. Along with this dynamic response, the regulatory mechanisms can also be dynamic over the time, leading to the flexibility of the transcriptional network topology. Additionally, the results also reflect the phenomenon that a gene can participate in various functions and thus be regulated by different sets of transcriptional regulators based on the context (e.g. TNF, MYD88).

## Discussion

### Functional characterization of inflammation-relevant pathways

Upon identification of four significant patterns of gene expression, a number of inflammation-specific pathways are selected by evaluating the enrichment of corresponding subsets in inflammation-specific pathways, including Toll-like receptor signaling, Cytokine-Cytokine receptor interaction, Apoptosis and JAK-STAT signaling cascade, etc. (**Table 1**). It is now well established that Toll like receptor signaling pathway is the first arm of the host defence system that is activated when endotoxin is recognized by pathogen recognition receptors [59]. During the recognition process, LPS binds and interacts with its signaling receptor (TLR4) which triggers a signal transduction cascade essential for the up-regulation of several pro-inflammatory mediators [60]. Such mediators including cytokines and chemokines interact with their appropriate receptors, giving rise to the Cytokine-Cytokine receptor signaling pathway that amplifies and propagates the inflammatory reaction throughout the cell until the system restores homeostasis [61]. Therefore, both Toll like receptor signaling and Cytokine-Cytokine receptor interaction pathways play a pivotal role in the pro-inflammatory response. Complementary to this, considerable attention has been given to the role of an excessive death of immune effector cells (apoptotic cells) during the progression of an aberrant inflammatory response [62]. The nature of apoptosis as a rectifying process has led researchers to the realization that identifying mediators that are critical in regulating the apoptotic-inflammatory imbalance might prove beneficial in treating human sepsis [63]. It is therefore reasonable to assume that apoptosis also plays a critical role in the endotoxin-induced inflammatory process. Along similar lines, JAK-STAT cascade is another highly enriched inflammation-specific pathway that exerts anti-inflammatory properties. Accordingly, recent data provide evidence that a STAT pathway from a receptor signaling system is a major determinant of key regulatory systems including feedback loops such as SOCS induction which subsequently suppresses the early induced Toll like receptor and cytokine signaling [64], [65]. Endotoxin–induced inflammation also causes a widespread suppression at the transcriptional response level of genes involved in mitochondrial energy production (Oxidative phosphorylation) and protein synthesis machinery (Ribosome). Such dysregulation in leukocyte bioenergetics together with persistent decrease in mitochondrial activity can lead to reduced cellular metabolism [66], resulting in the participation of a number of critical metabolic pathways, e.g. Citrate cycle, Pyrimidine and Pyruvate metabolism.

While comparing the inflammatory relevant pathways across expression patterns, we observe that there is a dynamic evolution of these pathways during the propagation of LPS signaling. For example, Toll like receptor signaling appears to be significantly enriched with a diverse array of genes that are early, middle or late up-regulated. From a biological standpoint, since the recognition process of LPS from its signaling receptor (TLR4) leads to the transcriptional activation of cytokines and chemokines (e.g. TNF, IL1B, CCL4), the early transcriptional event (t = 2 h) can possibly reflect this initiating process [67] while at later stages of the inflammatory reaction (t = 4 h), the effect of LPS has been translated into the signal transduction cascade mediated by the up-regulated cytokines. Such a signal transduction cascade is likely to be initiated by the conserved Toll/IL1 receptor (TIR) signaling domain [65] explaining the presence of TLR signaling in the early–up response (t = 2 h). Meanwhile, the presence of this pathway in the middle-up response (t = 4 h) is indicative for amplifying the activity of pro-inflammatory transcription factors (e.g. NFkB) essential for mediating the host response at later time events. Regarding the late up-regulated transcriptional event (t = 6 h), the system activates the expression of various genes that can effectively constraint the inflammatory response. Thus, anti-inflammatory mechanisms involve either the activation of JAK-STAT cascade (e.g. IL10RB, IL13RA1), or the increased expression of receptors (e.g. IL1R1, IL8RA, CCR1) that intend to replace and compensate for those that have been consumed during pro-inflammation [68]. Therefore, this cascade of events sheds significant insight on the dynamic evolution of critical pro-inflammatory pathways including TLR signaling, Apoptosis and Cytokine-Cytokine interaction signaling.

### Biological characterization of identified transcription factors

Predicated upon the hypothesis that subsets of co-expressed genes involved in the same biological pathway are more likely to be co-regulated, their transcriptional regulators are computationally predicted (**Table 2**). There is considerable evidence indicating the inflammatory relevance of the aforementioned inferred transcription factors including MEF2 [69], GATA [70], OCT1 [71], FKHD [72], ETSF [73], IRFF [74], NFKB [75] and CREB [76]. Specifically, the myocyte enhancer factor 2 (MEF2) transcription factor plays a central role in the transmission of extracellular signals to the genome and in the activation of genetic programs that control cell differentiation, proliferation, survival and apoptosis [77]. In addition to this, MEF2 proteins serve as the endpoints for multiple inflammatory signaling pathways including mitogen-activated protein kinase signaling pathway (MAPK) and thereby confer signal-responsiveness to downstream target genes [78]. Also, evidence [79], [80] suggests the dual role of MAPK signaling and thereby the activation of MEF2 transcription factor under TLR4 and Cytokine dependent mechanism. Furthermore, the octamer transcription factor −1 (OCT-1) has also been shown to function as a stress sensor modulating the activity of genes important for the cellular response to stress [81]. Although OCT-1 is a ubiquitous transcription factor, it has recently been demonstrated that it promotes cell survival signifying its involvement in the apoptosis signaling [82]. Additional studies [83] document the involvement of octamer binding transcription factors (OCT-1) in regulating the expression of TLR4 in humans; thus making it a critical regulator of Toll like receptor signaling. Furthermore, Forkhead Transcription Factors (FKHD) also play a major role in the control of apoptosis perhaps by affecting the transcription of the gene encoding FASL [84]. Since these regulators can be the substrate of the protein kinase B (Akt) preventing their nuclear translocation, it is expected that FKHD regulators promote cellular survival and thereby control the apoptotic machinery [85]. Moreover, IFN regulatory factors (IRFF) are a family of transcription factors that regulate expression of various pro-inflammatory and anti-inflammatory genes. Research findings reveal a critical role for these interferon regulatory proteins in the control of apoptosis [86], [87] while it has become evident [88], [89] that such regulators are also essential for TLR gene expression including the trans-acting factors, IRF-1 and IRF-2. This implies that in addition to up-regulation of pro-inflammatory gene expression, TLR stimulation also results in modulation of TLR gene expression itself via interferon transcription factors.

One of the most important cellular factors involved in the regulation of the host innate immune response is the nuclear factor (NF)-kB which can be activated by a variety of stimuli including bacterial products, inflammatory cytokines and growth factors [75], [90]. NF-kB is a pleiotropic transcription factor involved in the inducible expression of a diverse array of genes. As such, activation of the NF-kB signalling module involves not only the early up-regulation of pro-inflammatory cytokines but also the transcriptional control of apoptosis [91]. Oftentimes, transcriptional regulation requires the participation of several transcriptional factors through protein-protein interactions, known as transcriptional co-activators or co-repressors. For example, NF-kB encompasses an important family of inducible transcriptional activators critical in the regulation of the gene expression in response to injury and inflammatory stimuli. As such, the CREB-binding protein has been identified as co-activator of the NF-kB component p65 and might play an important role in the cytokine-induced expression of various immune and inflammatory genes [92]. Such observations emphasize the role of the CREB regulator in pro-inflammatory signaling pathways including TLR signaling pathway. Further evidence [93] confers the involvement of over-expressed CREB in inducing apoptosis while the control of FASL induction which mediates programmed cell death in human T lymphocytes [94] appears to be accomplished through a series of regulatory interactions that implicate the role of NF-kB and CREB/ATF pathways [95].

**Figure 4:**
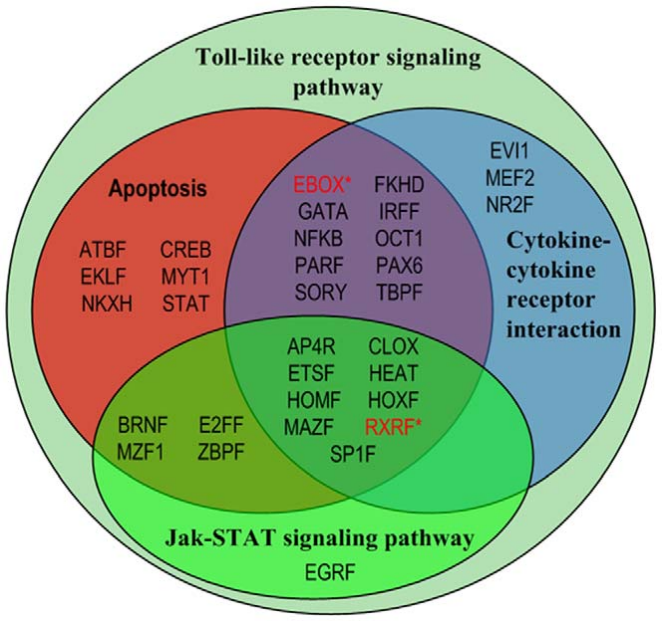
Pleiotropic effects of transcription factors across biological processes. Venn diagram shows pair-wise transcription factor combinations that overlap between the inflammatory relevant pathways (*: not present as TFs that regulate Toll-like receptor signaling pathway in this case).

Additionally, there is considerable evidence indicating the role of the early growth response-1 (member of EGR family) in regulating endotoxin induced SOCS-1 transcription [96]. SOCS-1 has been identified as a critical regulator of both adaptive cytokine signaling and innate immune responses and therefore understanding its transcriptional regulation under inflammatory conditions will no doubt be critical in understanding its role in limiting inflammatory responses [97]. Interestingly, these results demonstrate an important role of regulatory members of EGR family in regulating the endotoxin induced activity of the SOCS-1 promoter; thereby validating its presence in our computational predictions. In addition to transcriptional regulation of the anti-inflammatory SOCS family, recent data [98] also discussed a critical role of IL10 signaling in SOCS-3 expression which provides for feedback attenuation of cytokine induced immune responses. Other studies [99] document that SP1 regulator may be a central mediator of IL10 induction and thereby it may also play a crucial role in cytokine homeostasis. Accordingly, our method captures the family of stimulating protein 1 (SP1F) as a putative regulator in late phase (resolution) of the inflammatory response. On the other hand, we also observe a significant overlap across various biological processes while comparing these sets of TFs but it is reasonable since transcription factors are characterized by pleiotropic effects [58] (Figure 4). TLR signaling appears to be the principal pathway that initiates the host response to endotoxin and via the cross-talk among other pathways (e.g. Apoptosis, JAK-STAT) amplifies and propagates the inflammatory reaction providing for complex non-linear responses [100].

### Dynamic transcriptional regulatory program

**Figure 5:**
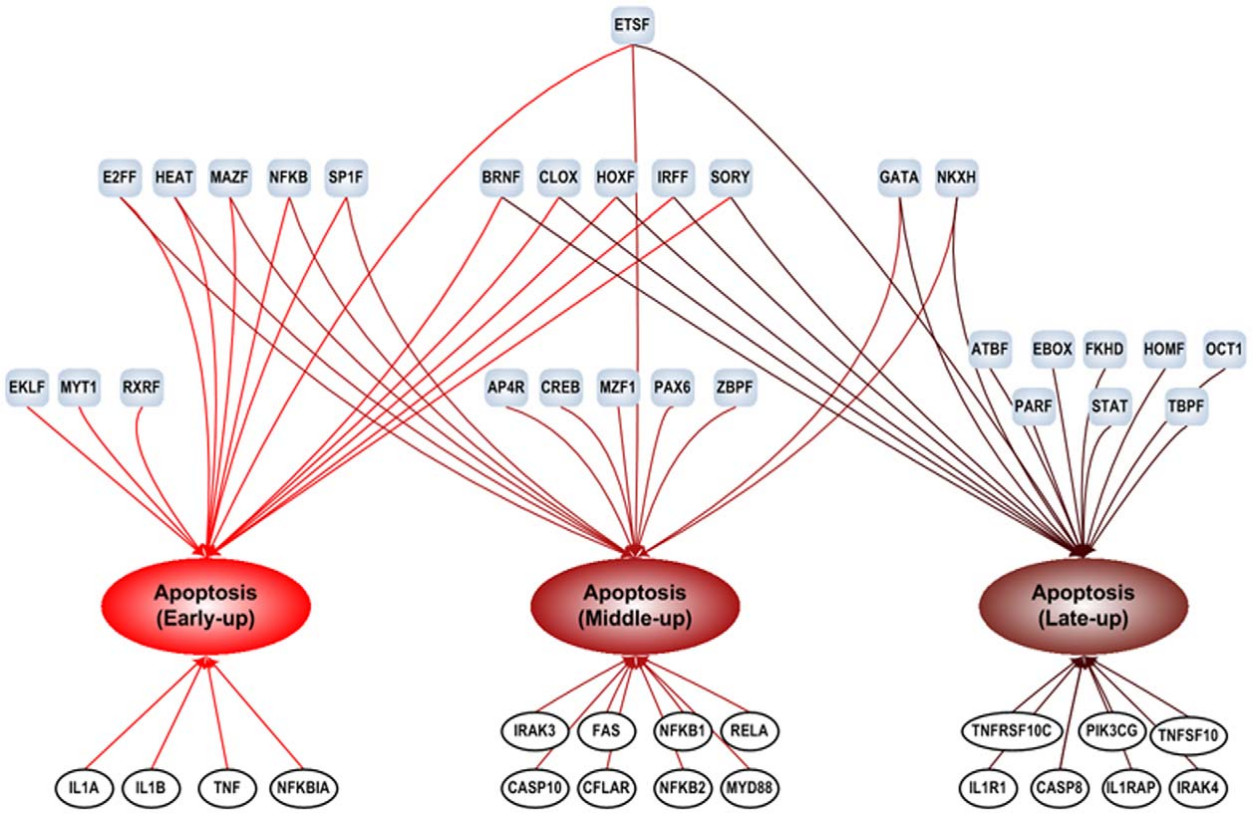
Dynamic representation of transcriptional regulatory network for apoptosis signaling. Transcription factors and target genes are shown as nodes and their putative regulatory interactions are drawn as edges.

The transcriptional regulatory program can potentially show a dynamic reorganization over time. In order to get a dynamic perspective, we focus on the apoptotic regulatory program as an illustration. Apoptosis is tightly regulated process that mainly responds to the initial stimulus followed by a cascade of events that involve the initiation and signal transduction phase. The initiation or preparation phase involves the activation of surface death receptors (extrinsic pathway); for example, TNFR responds to appropriate ligands (e.g. TNF), triggering downstream a signal transduction phase which eventually converges to the activation of effector caspases [101]. On the other hand, programmed cell death is regulated by caspase inhibitors which attenuate apoptosis. Such events would therefore reflect the early, middle and late apoptosis respectively (**Figure 5**).

Despite the identification of condition (time) specific regulators, the pleiotropic role of some transcription factors across multiple conditions is also observed. Accordingly, the myeloid transcription factor, MYT1, appears to be a critical pro-apoptotic regulator that affects the progression of the early stage apoptosis. Although considerable evidence [102], [103], [104] indicates the role of MYT1 in cell cycle regulation through induction of Cdc2 activity, further studies [105] also implicate the activation of cyclin dependent kinases in apoptosis induced by various stimuli. Therefore, such observation implies the possible involvement of Cdc2 regulators including MYT1 in enhancing programmed cell death during the early pro-inflammatory phase (early apoptosis). In addition to regulation of apoptosis, E2F family of DNA binding proteins has also been shown to have the ability to induce not only cell cycle but also programmed cell death [106], [107] by regulating the activation of pro-apoptotic genes as seen in **Figure 5**. Such evidence allows the identification of transcription factors with multifunctional capabilities essential for modifying coupled cellular processes including cell cycle and apoptotic behavior [102].

The retinoid X receptors (RXR) play a critical role in apoptosis. These proteins act functionally as either homodimers or heterodimers with other members of the nuclear hormones receptor family such as retinoid acid receptors (RAR) and peroxisome proliferators activated receptor (PPAR), which are all associated with the regulation of inflammation [108]. In particular, the interplay between RXR and RAR receptors has been shown to induce apoptosis which validates its role as a time specific regulator of early apoptosis [109], [110]. Despite the conventional role of NF-kB as anti-apoptotic [111], our results indicate its putative role in mediating cell death by transcriptionally up-regulating early and middle pro-apoptotic genes. Such antagonistic duality for the regulatory role of NF-kB has been recently outlined in [112] signifying its involvement in a pro-apoptotic fashion by up-regulating the expression of Fas. Reduced Fas expression followed by a reduction in apoptosis was also observed upon endotoxin challenge in relA^−/−^ (deficient) rodents suggesting a novel pro-apoptotic function for this protein in Fas induced cell death [113].

Putative regulators of middle and late apoptosis, including both pro- and anti-apoptotic transcription factors have been identified which aim at enhancing and controlling programmed cell death respectively. As such, activating binding protein (AP4R) which according to our results is present in middle apoptosis, has been shown to regulate the expression level of various caspases [114]. On the other hand, CREB binding protein has been shown to play a pivotal role in rescuing from apoptosis promoting cell survival via the induction of anti-apoptotic proteins [115], [116] while evidence [117] documents GATA as a novel MAPK substrate that plays an essential role in a cytokine mediated anti-apoptotic response. Based on our predictions GATA appears to be a significant regulator during late apoptosis conferring its role as anti-apoptotic protein. Since the late transcriptional event has been previously identified as a critical anti-inflammatory component, it is expected the regulators of late apoptosis to participate in controlling apoptosis by transcriptionally activating anti-apoptotic genes such as PIK3CG [118]. For example, studies on the STAT regulator have shown that it exerts an anti-apoptotic function required for maintenance of neutrophil homeostasis [119]. Furthermore, FKHD proteins also negatively affect apoptosis signaling through Akt signaling that is implicated in promoting cell survival [85], [120]. Both STAT and FKHD binding proteins exert direct regulatory links on the late apoptosis (**Figure 5**).

On the other hand, regarding the case that genes are involved in different functions within a particular expression pattern, a possibility relevant to alternative promoter usage can be suggested [50], [51]. Besides alternative promoters activated upon tissue-specific or condition-specific context, it is possible to hypothesize that they can be activated upon the coming set of biosignals which are transcriptional regulators, leading to the case that different protein isoforms are translated and thus the gene can be involved in different functions. Taken these together, our analysis provides significant insights on the potential regulatory interactions among transcriptional factors and their target genes which is a crucial step towards quantitative modelling of transcriptional regulatory networks [121].

In order to assess whether coexpressed genes are more likely to be coregulated, we estimate p-values of CRMs in individual gene batteries vs. the corresponding entire pattern of expression (**Table 3**). The results show that the estimated p-values values are similar to those calculated for the background set, implying that the entire pattern of coexpressed genes behaves more likely the same as a random background rather than as a set of genes that share a common regulatory mechanism (see Data S1, sheet ‘CRMs’ and ‘Middle-up TLR’). This supports our assumption related to the definition of a gene battery. Such preliminary results indicate that genes that are both coexpressed and functionally relevant are very likely to be governed by an underlying transcriptional regulatory program.

**Table 3:** Statistical significance of selected *cis*-regulatory modules*.

No.	*cis*- regulatory modules	avglen-minlen-maxlen	Common levels	vs. the background^1^(p-value^3^)	vs. the entire pattern^2^(p-value^3^)
1	+AP4R__−GATA__−HEAT$	288__169__485	0.75	1.88E-06	1.78E-05
2	+E2FF__+MOKF__−E2FF	333.8__170__514	0.75	1.06E-05	9.32E-08
3	+MOKF__−MZF1	168.7__95__236	0.75	3.36E-05	6.37E-07
4	+SP1F__−ETSF__−NFKB	189__110__268	0.75	3.58E-05	1.78E-05
5	+PAX6__+SNAP	154.2__66__260	0.75	4.29E-05	3.82E-05
6	+MOKF__−NKXH	101.3__37__194	0.875	4.51E-05	2.57E-05
7	+PAX6__−ETSF__−ZBPF	271.7__191__326	0.75	4.52E-05	3.82E-05
8	+NKXH__−CREB__−E2FF	518.3__403__788	0.75	6.85E-05	1.35E-04
9	+MAZF__−E2FF	72.1__32__98	0.875	9.82E-05	6.96E-05
10	+NFKB__−CREB__−SP1F	246.2__117__529	0.75	9.91E-05	1.35E-04

*: common significant *cis*-regulatory modules that are considered as transcriptional regulators for 8 genes in the middle-up expression pattern that belong to the apoptosis pathway;

‘+’|‘−’ TFBSs present on the forward | backward strand orientation;

$ : this CRM contains 3 TFBSs, binding sites of AP4R on the forward and of GATA, HEAT on the backward strand. Its average length is 288 bases while the minimum one has 169 bases and the maximum one has 485 bases. There are 8*0.75 = 6 instances of this CRM over 8 control regions of 8 genes;

1 : the background consists of 5,000 randomly selected genes;

2 : the entire corresponding pattern of gene expression (88 genes in this case);

3 : hyper-geometric p-value of this group vs. the background set or vs. the entire pattern.

### Predicting transcriptional regulators of an in vitro endotoxemia model

In order to assess the stability of our prediction, we applied the analysis to an in vitro human endotoxin model. Data are extracted from a culture of peripheral-blood-derived mononuclear cells stimulated by a high dose of LPS (100 ng/ml) [122]. Clustering approach reveals that there exist five critical transcriptional responses. Three of them characterize inflammatory phases similar to those identified in the analysis of *in vivo* data including an early-up response (284 probesets), a late-up response (700 probesets), and a down regulation (226 probesets). Due to a high dose of LPS administration, it would have expected an up- (367 probesets) and a down-regulation (319 probesets) without returning to the base line after 24 hr of LPS administration. Subsequently, a similar analysis of pathway enrichment (using KEGG database) was applied for each set of genes characterizing a transcriptional response. In an overlap with the analysis of *in vivo* data, we select statistically inflammatory relevant significant pathways (p-value<0.05) that were selected from the analysis on the *in vivo* human endotoxemia model. Accordingly, nine sets of genes that belong to a specific pathway and a pattern of gene expression were extracted, corresponding to nine genes batteries used to determine critical transcriptional regulators relevant to the inflammatory response in this study (**Table 4**).

**Table 4:** Critical transcription factors identified from the *in vitro* endotoxin study.

No.	Patterns	Functions	Transcription factors^*^
1	Early-up	Apoptosis	CLOX, E2FF, EGRF, EKLF, ETSF, FKHD, HOXC, HOXF, IRFF, MAZF, NKXH, NOLF, OCT1, RXRF, SORY, SP1F, STAT, XBBF
2	Late-up	Apoptosis	CREB, EKLF, MAZF, NFKB, SORY, ZBPF
3	Early-up	Toll-like receptor signaling pathway	AP1R, CLOX, E2FF, EGRF, EKLF, ETSF, HOXC, IRFF, NFKB, NOLF, NR2F, OCT1, RXRF, SORY, SP1F, STAT, XBBF, ZBPF
4	Late-up	Toll-like receptor signaling pathway	ABDB, CLOX, ETSF, HOMF, HOXF, IRFF, NFKB, NKXH, RXRF, SORY, STAT, TBPF
5	Early-up	Cytokine-cytokine receptor interaction	CREB, ETSF, FKHD, HOXF, RXRF, STAT, TBPF
6	Late-up	Cytokine-cytokine receptor interaction	ABDB, HOXF, NR2F, OCT1, RXRF, SORY, STAT
7	Early-up	Jak-STAT signaling pathway	ABDB, AP1R, AP4R, E2FF, EGRF, EKLF, ETSF, FKHD, HOMF, HOXF, IRFF, MAZF, NKXH, RXRF, SORY, SP1F, STAT, TBPF, XBBF, ZBPF
8	Late-up	Jak-STAT signaling pathway	ABDB, AP1R, AP4R, CLOX, CREB, E2FF, ETSF, FKHD, HOMF, HOXC, HOXF, NKXH, NR2F, OCT1, RXRF, SORY, TBPF
9	Up-remained	Pyrimidine metabolism	AP4R, E2FF, EGRF, EKLF, ETSF, FKHD, HOXF, MAZF, NFKB, NKXH, NOLF, NR2F, RXRF, SP1F, XBBF, ZBPF

Subsequently, the proposed method has been applied to search for statistical significant CRMs which are decomposed into a list of TFBSs to infer associated TFs that may be functional transcription factors in the regulation of inflammatory transcriptional responses. In a similar manner with the *in vivo* analysis, TFs that are present with the high frequency among gene batteries (at least three times) are reported (**Table 4**). We identify 27 critical TFs of which more than 80% are present in the list of relevant transcriptional regulators found in the analysis of the *in vivo* data including AP4R, CLOX, CREB, E2FF, EGRF, EKLF, ETSF, FKHD, HOMF, HOXF, IRFF, MAZF, NFKB, NKXH, NR2F, OCT1, RXRF, SORY, SP1F, STAT, TBPF, ZBPF. Given that different dosing amounts of LPS have been applied in two experiments, there may be different genes involved in the response of the same function between the *in vivo-* and *in vitro-* model, resulting in different TFs involved in the transcriptional regulation of the same gene battery between two cases. However, the significant overlap between two final lists of predicted TFs relevant to inflammatory transcriptional responses provides promising implications of the predictive performance of the method. Therefore, the proposed framework appears to be a robust and valuable methodology to identify critical transcriptional regulators relevant to biological responses under external stimuli (see details in Data S2).

### Computational issues

Regarding the computational definition of CRMs, although taking structural constraints (e.g. the strand orientation, the order, and the distance between successive TF-binding sites) into account can reduce the number of false positive matches, a strict definition of structural constraints can increase the rate of false negative prediction for the existence of CRMs on promoter sequences. Usually there is a pre-defined selection in the length (or window size) and the distance variation between successive binding sites in the composition of CRMs. However, our analysis excludes these parameters since a distance variation of several bases or several hundreds of bases may be invariant in the cells. Instead, we estimate the CRM length based on the length of its instances present on the control regions of corresponding genes in order to select common CRMs and calculate their significance values vs. the background set. Therefore, there is only one adjustable parameter (p-value) which defines the significance level of the resulting CRMs. The statistical significance thresholds for CRMs are selected following the red curve in **Figure 2** which is an approximation of the blue curve. The fluctuation of the blue curve is in part due to the random selection of the gene sets as well as due to the round-up to an integral number of the common level δ (70%) compared to the gene-set size N (e.g. either N is 6 or 7, there is the same common level for those recognized CRMs – present on at least 5 genes in this case) (see Data S1, sheet ‘p-value’).

Predicated upon the context-specific nature of the problem as well as a number of other relevant issues (e.g. establishing the criteria to measure the performance of the prediction, building up testing datasets), testing predicted CRMs and/or relevant TFs as ‘true’ or ‘false’ remains a challenge [27]. Thus of critical importance is to evaluate and validate the computational results based on literature evidence and those experimentally verified if possible. In this study, we employed a CRM-searching approach, similar to FrameWorker method [28], to identify common CRMs in each gene battery. However, the most critical issue is that a large proportion of mammalian genes possess multiple transcription start sites (TSSs) and therefore multiple alternative promoters regulate gene expression in a context-specific manner [123], [124], [125]. For instance, in a recent study Singer et al. [126] developed and employed a custom microarray platform to show that there are nearly 35,000 alternative putative promoters present on around 7,000 human genes. As a result, the computational identification of CRMs becomes a combinatorial problem and oftentimes a daunting task due to the large number of alternative promoters of genes in the gene battery. For example, 7 genes that belong to Apoptosis pathway and late-up expression pattern can produce totally 5,600 combinatorial promoter sets; or 10 genes that are in Cytokine-cytokine pathway and late-up expression pattern can create 13,440 combinatorial promoter sets; while complexity further increases in the oxidative phosphorylation group (down expression pattern) characterized by 40 genes and 1,274,019,840 combinatorial promoter sets. Consequently, searching for common CRMs in all promoter combinations is computationally intense. Yet, our novelty heuristic can reduce these complexities into only one running time but still preserve the same result (see appendix, lemma 1). In a similar manner, the strategy of converting promoter sequences into promoter profiles also makes the estimation of the significance of common CRMs vs. a large background set more computationally tractable [56].

Since it is not clear how long the promoter length should be, our computational analysis extracts highly qualitatively defined promoters from Genomatix databases [26] including those with either an experimentally defined length or a default if there is no associated prior length information. This default length (500 bp upstream plus 100 bp downstream the TSSs) is also supported from a recent experiment known as genome-wide open chromatin map [127]. Additionally, we also examined how the promoter length affects the *in silico* inference of CRMs. Specifically, we count the number of relevant TFs that can be considered as transcriptional regulators for the group of 8 genes that belong to the middle-up expression pattern and the apoptosis pathway. For each specific length of extracted promoters (27 promoters that are relevant transcripts; 100*x upstream and 20*x downstream bases, x from 4 to 10), we applied the same procedure to search for statistically significant CRMs and then infer the list of relevant TFs. The results show that the number of relevant TFs increase linearly with respect to increasing promoter lengths (see Data S1, sheet ‘Promoter length’). Thus, including prior information of the promoter lengths is very important to provide reliable computational predictions.

Another important challenge in computationally identifying TFs is associated with the fact that transcription factors can bind to regions far from the TSSs. For example, the P53 factor is a well established regulator for the programmed cell death (apoptosis) [128], [129]; however such regulator is not identified as putative TF in the gene batteries relevant to apoptosis pathway. However, if we increase the promoter length up to approximately 1,000 bp P53 is identified within the statistically significant CRMs. This leads to the hypothesis that P53 might work in a cooperative manner with other TFs that bind to the distant promoter regions. Alternatively, it has been recognized that P53 can affect apoptosis via novel transcription-independent pathways although its role as a mediator of transcription is well established [130], [131], [132]. For instance, apoptosis can still occur when P53 mutants incapable of acting as transcription regulator are introduced [133], [134]. This indicates the possibility that P53 might not directly regulate the apoptotic gene batteries as identified from our analysis. Thus, computational missing P53 as a relevant TF may be a reasonable result rather than a limitation from our computational analysis; yet, it is still a question to us in this study. However, since our analysis only searches for CRMs on the proximal promoters of genes, it should be acknowledged that we may miss some relevant transcription factors that bind to the regions far from the TSSs as well as enhancers that regulate the transcriptional process.

Furthermore, we also analyzed the reasons why no statistically significant CRM is found in the down-regulated gene batteries of the oxidative phosphorylation pathway (so-called OXPHOS group). OXPHOS itself is composed of genes that are coexpressed across numerous datasets under different conditions [135], [136] and it was proposed as a group of genes that might share a common regulatory mechanism [137]. However, we did not detect any complex-specific arrangement of TFBSs although it is highly enriched by a number of common TFBSs even when the promoter lengths are increased up to 1,000 bp upstream. Although this conclusion is similar to the result of a previous study [137], we note that subunits of each complex in OXPHOS group tend to have tighter coexpression with subunits of the same complex than subunits of other complex which is also proposed and discussed extensively in [137]. Based on the assumption that genes characterized by tightly coordinated expression levels are more likely to share common regulatory elements (proposed and demonstrated in [52]), we assume that genes belonging to the same complex might share some common set of regulatory signals. Therefore, we applied the same procedure of finding statistically significant CRMs on the control regions of those subgroups of genes including complex I – 17 genes, complex III – 6 genes, complex IV – 4 genes, and complex V – 13 genes. Eventually, we identified statistically significant CRMs for each complex from which relevant transcriptional regulators can be inferred. As a result, from a promoter analysis standpoint we are highly confident that subunits of each complex in OXPHOS group are more likely to be under a common regulatory mechanism rather than all the genes in the entire group (see Data S1, sheet ‘OXPHOS’). However, from a computational standpoint this result raised another possibility related to whether a subset of genes within a gene battery can provide more statistically significant CRMs than the entire gene battery. Assuming that the possibility is correct, this raises two questions including: (i) what is an appropriate size of the subset as well as (ii) how genes in the subset are selected. In order to address this issue, we make a case-study by randomly selecting a subset of N genes within the OXPHOS group (N = 17, 6, 4, and 13 respectively) and search for significant CRMs. The process is repeated 100 times and the average of minimum significance p-values is calculated. Results show that for N = 4, the average of minimum p-values is comparable to the one with N genes randomly selected from the background set (**Figure 2**). Yet, for the other cases the average of minimum p-values is less significant than the ones from the background set, suggesting that random subsets of genes within a gene battery behave more or less similarly to the case from the background set. Certainly, some subsets can provide more significant CRMs than the entire gene battery but how to interpret those selected subsets and the corresponding results remains a challenge. Therefore, it should be emphasized that using prior biological knowledge might overcome some of these limitations.

Our analysis has attempted to reverse engineer the underlying regulatory network of the human blood leukocyte response to a prototypical inflammatory stimulus (endotoxin). Given the transcriptional profiling data of human blood leukocytes, an elementary set of temporal responses with putative transcriptional regulators have been identified. A key feature of the analysis is the exploration of the concept ‘gene battery’ which represents for a group of genes that are both co-expressed and functional relevant to identify inflammatory transcriptional regulators using a context-specific searching approach [38]. Novel heuristics regarding to challenging issues e.g. eukaryotic genes consist of multiple alternative promoters leading to a huge computational complexity are also proposed. In order to provide a systematically unbiased *in silico* approach, CRM structural constraints are also adjusted so that no parameter is required except for the statistical significance thresholds. Furthermore, our analysis also allows for the reconstruction of a dynamic temporal regulatory network, making it a critical enabler for improving our understanding of how the transcriptional machinery ‘program’ effectively regulates key cellular processes.

Although no single analysis can identify all transcriptional regulators involved in a response, it has been demonstrated that the proposed framework can identify critical TFs that are relevant to acute inflammatory responses. Despite the fact that many methods have been proposed in the literature to search for relevant transcriptional regulators, different approaches explore different biological assumptions resulting to different sets of putative TFs which may or may not significantly overlap each other. Since the true extent of all TFs involved in the regulation of a complex response under some external stimuli is unknown, these differences could not be interpreted as the high- or low- accuracy of the methods. Instead, all of found TFs may be involved in different processes of the response but because of the limitation of the hypotheses used by the methods, they may not be recognized by a certain approach.

Novel methods are still proposed using different analytical approaches but generally they can be categorized into two main directions including mRNA expression-based [138], [139], [140] and TF binding pattern-based methods [28], [29], [30], [31], [36]. The first direction somehow utilizes the fundamental hypothesis that the mRNA expression level of TFs is proportional to their protein concentration but this may not be appropriate especially in higher eukaryotes since TF activation is often regulated post-translationally and acts somewhat in an independent manner of expression level. Some methods also require multiple-condition data as the input which may not be applicable when practical data are only sampled under one condition/treatment [138], [139], [140]. In the meanwhile, a lot of methods following to the latter direction have been developed e.g. FrameWorker [28], CMA [29], CRÈME [30], ModuleMiner [31], CisModule [36], BioMoby [141] etc. of which ours is among them. These are not limited by the mRNA expression proportion hypothesis but they are limited by promoter identification, TF binding profiles, and the underlying assumption to select the input set of ‘co-regulated’ genes.

In this study, we therefore opt to extend an available computational tool, FrameWorker, to take into account the fact that genes of higher eukaryotes contain multiple alternative promoters exploring the rich information of the Genomatix database on promoters and TF binding profiles. The underlying assumption that coexpressed genes are more likely to share some common regulatory mechanism when they are functional-relevant has been explored to predict putative functional activation of TFs in a specific context. These factors make our method become incomparable or unnecessary to compare with available methods. However, given the future availability of more complete TF binding data and other resources, the method could be enhanced by integrating protein-protein interaction to refine selected CRMs or using other tools to support the selection of relevant functions e.g. Pathway-Express [142]. Since each single method or even each direction always contains its own limitations and advantages, one possibility in future improvements could be the development of a framework to obtain a consensus result under diverse underlying hypotheses from various outputs of different methods.

## Methods

### Human endotoxemia model and data collection

#### *In vivo* data

The data used in this study were generated as part of the Inflammation and Host Response to Injury Large Scale Collaborative Project funded by the USPHS, U54 GM621119 [11], [143]. Human subjects were injected intravenously with endotoxin (CC-RE, lot 2) at a dose of 2-ng/kg body weight (endotoxin treated subjects) or 0.9% sodium chloride (placebo treated subjects). Following lysis of erythrocytes and isolation of total RNA from leukocyte pellets [11], biotin-labelled cRNA was hybridized to the Hu133A and Hu133B arrays containing a total of 44,924 probesets for measuring the expression level of genes that can be either activated or repressed in response to endotoxin at 0 (before treatment), 2, 4, 6, 9, and 24 hr. Data are publicly available through the GEO Database (#GSE3284). ANOVA technique (p<10^−4^) was then applied to filter significantly differentially expressed probesets, resulting in 3,269 selected probesets [55]. Average expression profiles of probesets over replicates for each time-point were used as the final input data for further analyses [144]. The data have been appropriately de-identified, and appropriate IRB approval and informed, written consent were obtained by the glue grant investigators [11].

#### *In vitro* data

Isolated from peripheral blood mononuclear cells collected from three healthy humans, adherent monocytes were cultured for 10 days in RPMI medium 1640 (20% FBS/L-glutamine/20 mM Hepes/penicillin/streptomycin/50 ng/ml macrophage colony-stimulating factor) to generate peripheral-blood-derived mononuclear cells [122]. These mononuclear cells were stimulated by 100 ng/ml LPS (*Salmonella minnesota* R595 ultra pure LPS; List Biological Laboratories, Campbell, CA) and sampled at 0 (before stimulation), 2, 4, 8, and 24 hr. Total RNA was isolated with TRIzol (Invitrogen, Carlsbad, CA) and two samples for each time-point were analyzed using HG-U133 Plus2 Affymetrix GeneChips producing mRNA expression profiles of 54,675 probesets (#GSE5504). Fold change (fold = 2.5) was then applied to filter significantly differentially expressed probesets, resulting in 2,892 selected probesets. Average expression profiles of probesets over replicates for each time-point were used as the final input data for further analyses [144].

### Clustering and selection

Utilizing the concept of the agreement matrix (AM) in consensus clustering, we recently proposed a novel method to identify the core set of probesets that are most agreeable in the AM of which they belong to the same or different patterns of gene expression [55]. In order to produce the agreement matrix, a number of different clustering methods along with different metrics (Euclidean, Manhattan, and Pearson correlation) were used to reduce the bias and assumption of any specific clustering method. After identifying the core set of probesets, the AM is reduced correspondingly to those selected probesets and then the hierarchical clustering is applied on the reduced AM to produce a number of gene expression patterns. Subsequently, we applied a trivial-cluster removal procedure and obtain four significant patterns of gene expression which are shown to be critical in the dynamics of acute human inflammation.

### Problem definition

**Figure 6:**
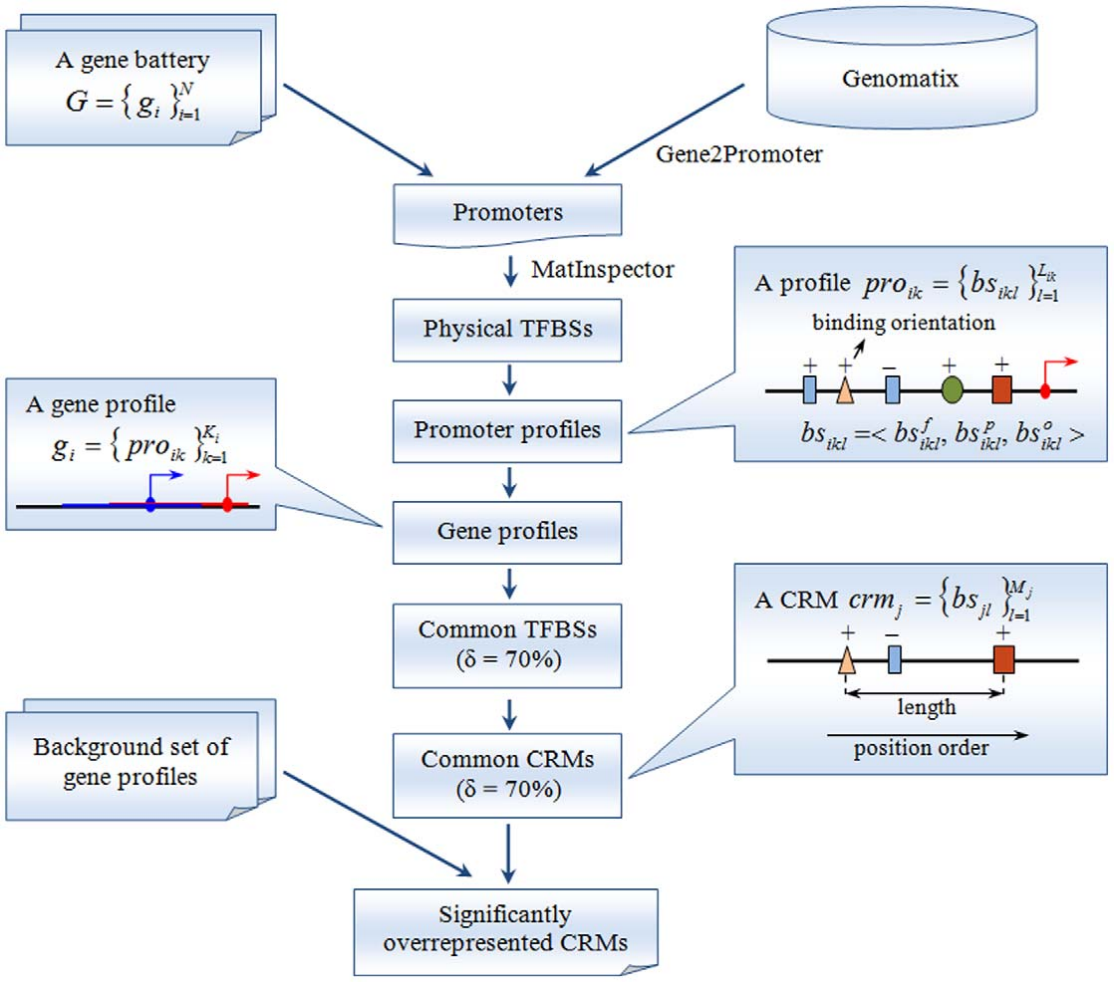
Flowchart of the CRM searching process. Each binding site is characterized by the TF name, position and binding strand orientation (+: forward and −: backward). Promoter sequences are converted into promoter profiles to speed up the calculation. A gene profile contains a set of promoter profiles that are corresponding to a set of alternative promoters of that gene. The background set contains 5,000 randomly selected human genes.

In this study, genes that are both coexpressed and functionally relevant are assumed to belong to the same ‘gene battery’. The problem of CRM searching can be formalized as follows: given a set of N putatively coregulated genes 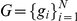, each of which contains K_i_ alternative promoters 
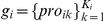
 whereas each promoter is represented by a list of L_ik_ binding sites (‘promoter profiles’) 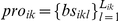 and each binding site is a 3-tuple of corresponding transcription factor name f, position p and binding orientation o: 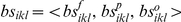, find a set of M *cis*-regulatory modules (CRMs) 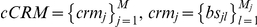 that are present as common over a threshold δ (70% in this study) on the set of gene promoters (M_j_ is the number of binding sites, yet to be determined, in CRM crm_j_). The statistical significance of each commonly recognized CRM vs. a background set of genes is then estimated selecting only significant CRMs. The subscripts i, k, l, j indicate the gene number, the promoter number, the binding site number, and the CRM number respectively. An illustration of the computational framework is presented in **Figure 6** while more details are discussed in the following section.

### Discovery of TFBSs and promoter profiles

Based on a comprehensive database of promoters – Genomatix [26], a set of transcript-relevant promoters are extracted coupled with multiple alternative promoters and experimental information about the promoter length including those with either an experimentally defined length or a default if there is no associated prior length information (500 bp upstream plus 100 bp downstream the TSSs). MatInspector [20] is then applied to scan for PWM matches on those promoter sequences using optimal parameters from MatBase [26]. In order to speed up the process of discovering CRMs as outlined in [56], each promoter is re-modelled with a list of L_ik_ TFBSs ordered by their local positions on the promoter sequences and represented by the corresponding TF name (e.g. NFKB, ETSF) along with the binding orientation 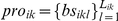. The conversion aims to answer two basic questions: (i) given a promoter sequence, identify whether a TFBS or a CRM is present on this promoter or not, and (ii) given a gene with K_i_ alternative promoters, determine if a TFBS or a CRM is present on any promoter sequence of this gene. From a computational standpoint each promoter profile is loaded into a hash table whose field ‘key’ includes the TFBS name plus the binding orientation (e.g. +ETSF, −PAX6, ‘+’ as forward and ‘−’ as backward binding orientation) and field ‘value’ is the position list of the corresponding TFBS with the same binding orientation. For example, if the key is ‘+ETSF’ and the corresponding value is ‘373__386’, we know that transcription factor ETSF is forward binding to the promoter at the local position −373 or −386 upstream. As a result, to decide the existence of a TFBS including the binding orientation on a promoter the process only makes a quick search in the hash keys. In a similar way, to determine the present of a CRM on a promoter the process will take into account the binding orientation from the keys and the positions from the values of corresponding keys to evaluate the structural constraints (see Data S1, sheet ‘Promoter profiles’).

### Common *cis*-regulatory modules

Computationally, a *cis*-regulatory module crm_j_ is a list of M_j_ non-overlapping TFBSs ordered by their positions on the promoter sequence and characterized with their corresponding binding strand orientation. For example, CRM ‘+NFKB__−CREB__−SP1F’ consists of three successive TFBSs of transcription factors NFKB, CREB, SP1F with the binding strand orientation forward, backward, and backward respectively. Besides the binding orientation and the position order of TFBSs, CRMs are also characterized by their length. If CRM A appears to be common in a gene battery of N genes, the average length of all instances of A on N genes is considered to be the length of this CRM. In the case that A presents more than one time on promoters of gene i, the length of instance A for this gene will be the minimum one. Subsequently, to estimate the common level of this CRM we only take into account those instances with the length approximate to the average one (e.g. from the half to the double). If the number of such instances over N is higher than a frequency threshold (δ = 70% in this study), CRM A is considered as a common CRM of the gene battery.

However, a gene can have multiple alternative promoters and virtually in all cases, it is not known which promoter of the gene is activated. To identify activation of putative promoters, one solution would be to search for all possible combinations of promoters in the gene set. Yet given a set of N genes, each gene with K alternative promoters in average, the total combinatorial number of promoter sets is K^N^ which is computationally intense and sometimes impossible to search for all promoter combinations. Consequently, we propose a novel heuristic *where if a TFBS or a CRM is present on any promoter sequence of a gene, it is considered as present on the control regions of that gene*. The heuristic results in one-time searching instead of K^N^ but still produces the same results as the brute-force search in all combinations of promoters (see Appendix S1, procedure ‘IsPresent’ in Algorithms S1 and Procedures S1). Using this heuristic, the main algorithm to search for common CRMs in a gene battery, similar to FrameWorker [28], can be simply described with two primary steps as follows: (1) identify all potential TFBSs that are common in a gene battery and (2) employ the breadth first search technique to search for all possible combination of all commonly found TFBSs in step 1, each of which is a potentially common CRM yet to be determined quickly by the heuristic above (see details in Algorithms S1 and Procedures S1).

### Estimating the statistical significance of *cis*-regulatory modules

Due to the fact that a CRM can be present on promoters of many genes in the background set, we estimate the statistical significance of commonly identified CRMs for each gene battery vs. the background set to select those that are significantly overrepresented. Based on promoter profiles, the procedure can directly identify whether a particular CRM is present on any promoter sequence of a gene in real-time despite the large background set of genes. This calculation provides a corresponding hyper-geometric p-value defined as follows:
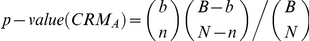
where B and b is the number of genes and the number of hits respectively in the background set which is made up of 5,000 randomly selected genes in human genome; N and n is the number of genes and hits in the gene battery, respectively.

## Supporting Information

Data S1 Provide detailed results for the analysis of *in vivo* data, including common significant CRMs recognized, the detailed data of Figure 2, the predicted relevant TFs found, the examples of promoter profiles, and the effects of promoter lengths on the number of relevant TFs found.
(XLS)

Data S2 Provide detailed results for the analysis of *in vitro* data, including critical transcriptional responses, selected gene batteries, common significant CRMs recognized, and predicted relevant TFs.
(XLS)

Algorithms S1 Restate the problem of discovery CRMs in gene batteries and provide detailed pseudo-code for algorithms, including the procedure ‘IsPresent’ and the algorithm of the main procedure.
(DOC)

Appendix S1 Provide an illustration to prove that the set of common CRMs found by the proposed heuristic is the same with the set of common CRMs found from all promoter combinations.
(DOC)

Procedures S1 Provide source codes of some main procedure (by Perl language) including (1) check the existence of a CRM on alternative promoters of a gene, (2) search for common CRMs in a gene battery, and (3) estimate the hyper-geometric p-value of CRMs vs. the background set.
(DOC)

## References

[1] Beutler B, Rietschel ET (2003). Innate immune sensing and its roots: the story of endotoxin.. Nat Rev Immunol.

[2] Opal SM, DePalo VA (2000). Anti-inflammatory cytokines.. Chest.

[3] Nathan C (2002). Points of control in inflammation.. Nature.

[4] Hotchkiss RS, Karl IE (2003). The pathophysiology and treatment of sepsis.. N Engl J Med.

[5] Tetta C, Fonsato V, Ronco C, Camussi G (2005). Recent insights into the pathogenesis of severe sepsis.. Crit Care Resusc.

[6] Copeland S, Warren HS, Lowry SF, Calvano SE, Remick D (2005). Acute inflammatory response to endotoxin in mice and humans.. Clin Diagn Lab Immunol.

[7] Lowry SF (2005). Human endotoxemia: a model for mechanistic insight and therapeutic targeting.. Shock.

[8] Van Zee KJ, Coyle SM, Calvano SE, Oldenburg HS, Stiles DM (1995). Influence of IL-1 receptor blockade on the human response to endotoxemia.. J Immunol.

[9] van Deventer SJ, Buller HR, ten Cate JW, Aarden LA, Hack CE (1990). Experimental endotoxemia in humans: analysis of cytokine release and coagulation, fibrinolytic, and complement pathways.. Blood.

[10] Santos AA, Wilmore DW (1996). The systemic inflammatory response: perspective of human endotoxemia.. Shock.

[11] Calvano SE, Xiao W, Richards DR, Felciano RM, Baker HV (2005). A network-based analysis of systemic inflammation in humans.. Nature.

[12] Phillips T (2008). Regulation of transcription and gene expression in eukaryotes.. Nature Education.

[13] Lemon B, Tjian R (2000). Orchestrated response: a symphony of transcription factors for gene control.. Genes Dev.

[14] Levine M, Tjian R (2003). Transcription regulation and animal diversity.. Nature.

[15] Maston GA, Evans SK, Green MR (2006). Transcriptional regulatory elements in the human genome.. Annu Rev Genomics Hum Genet.

[16] Kim TH, Ren B (2006). Genome-wide analysis of protein-DNA interactions.. Annu Rev Genomics Hum Genet.

[17] Ren B, Robert F, Wyrick JJ, Aparicio O, Jennings EG (2000). Genome-wide location and function of DNA binding proteins.. Science.

[18] Djordjevic M (2007). SELEX experiments: new prospects, applications and data analysis in inferring regulatory pathways.. Biomol Eng.

[19] Stoltenburg R, Reinemann C, Strehlitz B (2007). SELEX–a (r)evolutionary method to generate high-affinity nucleic acid ligands.. Biomol Eng.

[20] Cartharius K, Frech K, Grote K, Klocke B, Haltmeier M (2005). MatInspector and beyond: promoter analysis based on transcription factor binding sites.. Bioinformatics.

[21] Chekmenev DS, Haid C, Kel AE (2005). P-Match: transcription factor binding site search by combining patterns and weight matrices.. Nucleic Acids Res.

[22] Fessele S, Maier H, Zischek C, Nelson PJ, Werner T (2002). Regulatory context is a crucial part of gene function.. Trends Genet.

[23] Harbison CT, Gordon DB, Lee TI, Rinaldi NJ, Macisaac KD (2004). Transcriptional regulatory code of a eukaryotic genome.. Nature.

[24] Balmer JE, Blomhoff R (2006). Anecdotes, data and regulatory modules.. Biol Lett.

[25] Davidson EH (2001). Genomic Regulatory Systems: Development and Evolution.

[26] Genomatix http://www.genomatix.de

[27] Klepper K, Sandve GK, Abul O, Johansen J, Drablos F (2008). Assessment of composite motif discovery methods.. BMC Bioinformatics.

[28] Frech K, Danescu-Mayer J, Werner T (1997). A novel method to develop highly specific models for regulatory units detects a new LTR in GenBank which contains a functional promoter.. J Mol Biol.

[29] Waleev T, Shtokalo D, Konovalova T, Voss N, Cheremushkin E (2006). Composite Module Analyst: identification of transcription factor binding site combinations using genetic algorithm.. Nucleic Acids Res.

[30] Sharan R, Ben-Hur A, Loots GG, Ovcharenko I (2004). CREME: Cis-Regulatory Module Explorer for the human genome.. Nucleic Acids Res.

[31] Van Loo P, Aerts S, Thienpont B, De Moor B, Moreau Y (2008). ModuleMiner - improved computational detection of cis-regulatory modules: are there different modes of gene regulation in embryonic development and adult tissues?. Genome Biol.

[32] Sinha S, Liang Y, Siggia E (2006). Stubb: a program for discovery and analysis of cis-regulatory modules.. Nucleic Acids Res.

[33] Chan BY, Kibler D (2005). Using hexamers to predict cis-regulatory motifs in Drosophila.. BMC Bioinformatics.

[34] Taylor J, Tyekucheva S, King DC, Hardison RC, Miller W (2006). ESPERR: learning strong and weak signals in genomic sequence alignments to identify functional elements.. Genome Res.

[35] Wang H, Zhang Y, Cheng Y, Zhou Y, King DC (2006). Experimental validation of predicted mammalian erythroid cis-regulatory modules.. Genome Res.

[36] Zhou Q, Wong WH (2004). CisModule: de novo discovery of cis-regulatory modules by hierarchical mixture modeling.. Proc Natl Acad Sci U S A.

[37] Ivan A, Halfon MS, Sinha S (2008). Computational discovery of cis-regulatory modules in Drosophila without prior knowledge of motifs.. Genome Biol.

[38] Nguyen TT, Androulakis IP (2009). Recent Advances in the Computational Discovery of Transcription Factor Binding Sites.. Algorithms.

[39] Britten RJ, Davidson EH (1969). Gene regulation for higher cells: a theory.. Science.

[40] Berman BP, Nibu Y, Pfeiffer BD, Tomancak P, Celniker SE (2002). Exploiting transcription factor binding site clustering to identify cis-regulatory modules involved in pattern formation in the Drosophila genome.. Proc Natl Acad Sci U S A.

[41] Halfon MS, Grad Y, Church GM, Michelson AM (2002). Computation-based discovery of related transcriptional regulatory modules and motifs using an experimentally validated combinatorial model.. Genome Res.

[42] Rajewsky N, Vergassola M, Gaul U, Siggia ED (2002). Computational detection of genomic cis-regulatory modules applied to body patterning in the early Drosophila embryo.. BMC Bioinformatics.

[43] Segal E, Shapira M, Regev A, Pe'er D, Botstein D (2003). Module networks: identifying regulatory modules and their condition-specific regulators from gene expression data.. Nat Genet.

[44] Nelander S, Larsson E, Kristiansson E, Mansson R, Nerman O (2005). Predictive screening for regulators of conserved functional gene modules (gene batteries) in mammals.. BMC Genomics.

[45] Altman RB, Raychaudhuri S (2001). Whole-genome expression analysis: challenges beyond clustering.. Curr Opin Struct Biol.

[46] Roth FP, Hughes JD, Estep PW, Church GM (1998). Finding DNA regulatory motifs within unaligned noncoding sequences clustered by whole-genome mRNA quantitation.. Nat Biotechnol.

[47] Tavazoie S, Hughes JD, Campbell MJ, Cho RJ, Church GM (1999). Systematic determination of genetic network architecture.. Nat Genet.

[48] Elkon R, Linhart C, Sharan R, Shamir R, Shiloh Y (2003). Genome-wide in silico identification of transcriptional regulators controlling the cell cycle in human cells.. Genome Res.

[49] Long F, Liu H, Hahn C, Sumazin P, Zhang MQ (2004). Genome-wide prediction and analysis of function-specific transcription factor binding sites.. In Silico Biol.

[50] Allocco DJ, Kohane IS, Butte AJ (2004). Quantifying the relationship between co-expression, co-regulation and gene function.. BMC Bioinformatics.

[51] Brown CD, Johnson DS, Sidow A (2007). Functional architecture and evolution of transcriptional elements that drive gene coexpression.. Science.

[52] Choi D, Fang Y, Mathers WD (2006). Condition-specific coregulation with cis-regulatory motifs and modules in the mouse genome.. Genomics.

[53] Huang R, Wallqvist A, Covell DG (2006). Comprehensive analysis of pathway or functionally related gene expression in the National Cancer Institute's anticancer screen.. Genomics.

[54] Hannenhalli S, Levy S (2003). Transcriptional regulation of protein complexes and biological pathways.. Mamm Genome.

[55] Nguyen TT, Nowakowski RS, Androulakis IP (2009). Unsupervised selection of highly coexpressed and noncoexpressed genes using a consensus clustering approach.. Omics.

[56] Schones DE, Smith AD, Zhang MQ (2007). Statistical significance of cis-regulatory modules.. BMC Bioinformatics.

[57] Hutton JJ, Jegga AG, Kong S, Gupta A, Ebert C (2004). Microarray and comparative genomics-based identification of genes and gene regulatory regions of the mouse immune system.. BMC Genomics.

[58] Rodriguez-Caso C, Medina MA, Sole RV (2005). Topology, tinkering and evolution of the human transcription factor network.. Febs J.

[59] Aderem A, Smith KD (2004). A systems approach to dissecting immunity and inflammation.. Semin Immunol.

[60] Takeda K, Akira S (2005). Toll-like receptors in innate immunity.. Int Immunol.

[61] Frankenstein Z, Alon U, Cohen IR (2006). The immune-body cytokine network defines a social architecture of cell interactions.. Biol Direct.

[62] Hotchkiss RS, Nicholson DW (2006). Apoptosis and caspases regulate death and inflammation in sepsis.. Nat Rev Immunol.

[63] Wesche-Soldato DE, Swan RZ, Chung CS, Ayala A (2007). The apoptotic pathway as a therapeutic target in sepsis.. Curr Drug Targets.

[64] Murray PJ (2007). The JAK-STAT signaling pathway: input and output integration.. J Immunol.

[65] Barton GM, Medzhitov R (2003). Toll-like receptor signaling pathways.. Science.

[66] Singer M, Brealey D (1999). Mitochondrial dysfunction in sepsis.. Biochem Soc Symp.

[67] Kaczorowski DJ, Mollen KP, Edmonds R, Billiar TR (2008). Early events in the recognition of danger signals after tissue injury.. J Leukoc Biol.

[68] Liu Y, Wang Y, Yamakuchi M, Isowaki S, Nagata E (2001). Upregulation of toll-like receptor 2 gene expression in macrophage response to peptidoglycan and high concentration of lipopolysaccharide is involved in NF-kappa b activation.. Infect Immun.

[69] Han J, Jiang Y, Li Z, Kravchenko VV, Ulevitch RJ (1997). Activation of the transcription factor MEF2C by the MAP kinase p38 in inflammation.. Nature.

[70] Shapira M, Hamlin BJ, Rong J, Chen K, Ronen M (2006). A conserved role for a GATA transcription factor in regulating epithelial innate immune responses.. Proc Natl Acad Sci U S A.

[71] Serfling E, Avots A, Neumann M (1995). The architecture of the interleukin-2 promoter: a reflection of T lymphocyte activation.. Biochim Biophys Acta.

[72] Coffer PJ, Burgering BM (2004). Forkhead-box transcription factors and their role in the immune system.. Nat Rev Immunol.

[73] Gallant S, Gilkeson G (2006). ETS transcription factors and regulation of immunity.. Arch Immunol Ther Exp (Warsz).

[74] Taniguchi T (1997). Transcription factors IRF-1 and IRF-2: linking the immune responses and tumor suppression.. J Cell Physiol.

[75] Tak PP, Firestein GS (2001). NF-kappaB: a key role in inflammatory diseases.. J Clin Invest.

[76] McKay LI, Cidlowski JA (2000). CBP (CREB binding protein) integrates NF-kappaB (nuclear factor-kappaB) and glucocorticoid receptor physical interactions and antagonism.. Mol Endocrinol.

[77] Potthoff MJ, Olson EN (2007). MEF2: a central regulator of diverse developmental programs.. Development.

[78] Olson EN (2004). Undermining the endothelium by ablation of MAPK-MEF2 signaling.. J Clin Invest.

[79] Maung AA, Fujimi S, Miller ML, MacConmara MP, Mannick JA (2005). Enhanced TLR4 reactivity following injury is mediated by increased p38 activation.. J Leukoc Biol.

[80] Kawai K, Saito A, Sudo T, Osada H (2008). Specific regulation of cytokine-dependent p38 MAP kinase activation by p62/SQSTM1.. J Biochem.

[81] Tantin D, Schild-Poulter C, Wang V, Hache RJ, Sharp PA (2005). The octamer binding transcription factor Oct-1 is a stress sensor.. Cancer Res.

[82] Schild-Poulter C, Shih A, Tantin D, Yarymowich NC, Soubeyrand S (2007). DNA-PK phosphorylation sites on Oct-1 promote cell survival following DNA damage.. Oncogene.

[83] Rehli M, Poltorak A, Schwarzfischer L, Krause SW, Andreesen R (2000). PU.1 and interferon consensus sequence-binding protein regulate the myeloid expression of the human Toll-like receptor 4 gene.. J Biol Chem.

[84] Kitada S, Pedersen IM, Schimmer AD, Reed JC (2002). Dysregulation of apoptosis genes in hematopoietic malignancies.. Oncogene.

[85] Brunet A, Bonni A, Zigmond MJ, Lin MZ, Juo P (1999). Akt promotes cell survival by phosphorylating and inhibiting a Forkhead transcription factor.. Cell.

[86] Cuesta N, Nhu QM, Zudaire E, Polumuri S, Cuttitta F (2007). IFN regulatory factor-2 regulates macrophage apoptosis through a STAT1/3- and caspase-1-dependent mechanism.. J Immunol.

[87] Cuesta N, Salkowski CA, Thomas KE, Vogel SN (2003). Regulation of lipopolysaccharide sensitivity by IFN regulatory factor-2.. J Immunol.

[88] Hertzog PJ, O'Neill LA, Hamilton JA (2003). The interferon in TLR signaling: more than just antiviral.. Trends Immunol.

[89] Nhu QM, Cuesta N, Vogel SN (2006). Transcriptional regulation of lipopolysaccharide (LPS)-induced Toll-like receptor (TLR) expression in murine macrophages: role of interferon regulatory factors 1 (IRF-1) and 2 (IRF-2).. J Endotoxin Res.

[90] Tripathi P, Aggarwal A (2006). NF-kB transcription factor: a key player in the generation of immune response.. Current Science.

[91] Ward C, Chilvers ER, Lawson MF, Pryde JG, Fujihara S (1999). NF-kappaB activation is a critical regulator of human granulocyte apoptosis in vitro.. J Biol Chem.

[92] Gerritsen ME, Williams AJ, Neish AS, Moore S, Shi Y (1997). CREB-binding protein/p300 are transcriptional coactivators of p65.. Proc Natl Acad Sci U S A.

[93] Saeki K, Yuo A, Suzuki E, Yazaki Y, Takaku F (1999). Aberrant expression of cAMP-response-element-binding protein (‘CREB’) induces apoptosis.. Biochem J.

[94] Alderson MR, Tough TW, Davis-Smith T, Braddy S, Falk B (1995). Fas ligand mediates activation-induced cell death in human T lymphocytes.. J Exp Med.

[95] Chen X, Zachar V, Zdravkovic M, Guo M, Ebbesen P (1997). Role of the Fas/Fas ligand pathway in apoptotic cell death induced by the human T cell lymphotropic virus type I Tax transactivator.. J Gen Virol.

[96] Mostecki J, Showalter BM, Rothman PB (2005). Early growth response-1 regulates lipopolysaccharide-induced suppressor of cytokine signaling-1 transcription.. J Biol Chem.

[97] Ilangumaran S, Rottapel R (2003). Regulation of cytokine receptor signaling by SOCS1.. Immunol Rev.

[98] Qin H, Roberts KL, Niyongere SA, Cong Y, Elson CO (2007). Molecular mechanism of lipopolysaccharide-induced SOCS-3 gene expression in macrophages and microglia.. J Immunol.

[99] Brightbill HD, Plevy SE, Modlin RL, Smale ST (2000). A prominent role for Sp1 during lipopolysaccharide-mediated induction of the IL-10 promoter in macrophages.. J Immunol.

[100] Natarajan M, Lin KM, Hsueh RC, Sternweis PC, Ranganathan R (2006). A global analysis of cross-talk in a mammalian cellular signalling network.. Nat Cell Biol.

[101] Thornberry NA, Lazebnik Y (1998). Caspases: enemies within.. Science.

[102] Fussenegger M, Bailey JE (1998). Molecular regulation of cell-cycle progression and apoptosis in mammalian cells: implications for biotechnology.. Biotechnol Prog.

[103] Liu F, Rothblum-Oviatt C, Ryan CE, Piwnica-Worms H (1999). Overproduction of human Myt1 kinase induces a G2 cell cycle delay by interfering with the intracellular trafficking of Cdc2-cyclin B1 complexes.. Mol Cell Biol.

[104] Liu F, Stanton JJ, Wu Z, Piwnica-Worms H (1997). The human Myt1 kinase preferentially phosphorylates Cdc2 on threonine 14 and localizes to the endoplasmic reticulum and Golgi complex.. Mol Cell Biol.

[105] Zhou BB, Li H, Yuan J, Kirschner MW (1998). Caspase-dependent activation of cyclin-dependent kinases during Fas-induced apoptosis in Jurkat cells.. Proc Natl Acad Sci U S A.

[106] Bell LA, Ryan KM (2004). Life and death decisions by E2F-1.. Cell Death Differ.

[107] Furukawa Y, Nishimura N, Furukawa Y, Satoh M, Endo H (2002). Apaf-1 is a mediator of E2F-1-induced apoptosis.. J Biol Chem.

[108] Kuenzli S, Tran C, Saurat JH (2004). Retinoid receptors in inflammatory responses: a potential target for pharmacology.. Curr Drug Targets Inflamm Allergy.

[109] Szondy Z, Reichert U, Fesus L (1998). Retinoic acids regulate apoptosis of T lymphocytes through an interplay between RAR and RXR receptors.. Cell Death Differ.

[110] Piedrafita FJ, Pfahl M (1997). Retinoid-induced apoptosis and Sp1 cleavage occur independently of transcription and require caspase activation.. Mol Cell Biol.

[111] Beg AA, Baltimore D (1996). An essential role for NF-kappaB in preventing TNF-alpha-induced cell death.. Science.

[112] Radhakrishnan SK, Kamalakaran S (2006). Pro-apoptotic role of NF-kappaB: implications for cancer therapy.. Biochim Biophys Acta.

[113] Ouaaz F, Li M, Beg AA (1999). A critical role for the RelA subunit of nuclear factor kappaB in regulation of multiple immune-response genes and in Fas-induced cell death.. J Exp Med.

[114] Tsujimoto K, Ono T, Sato M, Nishida T, Oguma T (2005). Regulation of the expression of caspase-9 by the transcription factor activator protein-4 in glucocorticoid-induced apoptosis.. J Biol Chem.

[115] Wilson BE, Mochon E, Boxer LM (1996). Induction of bcl-2 expression by phosphorylated CREB proteins during B-cell activation and rescue from apoptosis.. Mol Cell Biol.

[116] Ruvolo PP, Deng X, May WS (2001). Phosphorylation of Bcl2 and regulation of apoptosis.. Leukemia.

[117] Yu YL, Chiang YJ, Chen YC, Papetti M, Juo CG (2005). MAPK-mediated phosphorylation of GATA-1 promotes Bcl-XL expression and cell survival.. J Biol Chem.

[118] Hirsch E, Costa C, Ciraolo E (2007). Phosphoinositide 3-kinases as a common platform for multi-hormone signaling.. J Endocrinol.

[119] Fievez L, Desmet C, Henry E, Pajak B, Hegenbarth S (2007). STAT5 is an ambivalent regulator of neutrophil homeostasis.. PLoS ONE.

[120] Kennedy SG, Wagner AJ, Conzen SD, Jordan J, Bellacosa A (1997). The PI 3-kinase/Akt signaling pathway delivers an anti-apoptotic signal.. Genes Dev.

[121] Seok J, Xiao W, Moldawer LL, Davis RW, Covert MW (2009). A dynamic network of transcription in LPS-treated human subjects.. BMC Syst Biol.

[122] Roach JC, Smith KD, Strobe KL, Nissen SM, Haudenschild CD (2007). Transcription factor expression in lipopolysaccharide-activated peripheral-blood-derived mononuclear cells.. Proc Natl Acad Sci U S A.

[123] Davuluri RV, Suzuki Y, Sugano S, Plass C, Huang TH (2008). The functional consequences of alternative promoter use in mammalian genomes.. Trends Genet.

[124] Kapranov P, Willingham AT, Gingeras TR (2007). Genome-wide transcription and the implications for genomic organization.. Nat Rev Genet.

[125] Sandelin A, Carninci P, Lenhard B, Ponjavic J, Hayashizaki Y (2007). Mammalian RNA polymerase II core promoters: insights from genome-wide studies.. Nat Rev Genet.

[126] Singer GA, Wu J, Yan P, Plass C, Huang TH (2008). Genome-wide analysis of alternative promoters of human genes using a custom promoter tiling array.. BMC Genomics.

[127] Boyle AP, Davis S, Shulha HP, Meltzer P, Margulies EH (2008). High-resolution mapping and characterization of open chromatin across the genome.. Cell.

[128] Fridman JS, Lowe SW (2003). Control of apoptosis by p53.. Oncogene.

[129] Vousden KH, Lu X (2002). Live or let die: the cell's response to p53.. Nat Rev Cancer.

[130] Chipuk JE, Kuwana T, Bouchier-Hayes L, Droin NM, Newmeyer DD (2004). Direct activation of Bax by p53 mediates mitochondrial membrane permeabilization and apoptosis.. Science.

[131] Ding HF, Lin YL, McGill G, Juo P, Zhu H (2000). Essential role for caspase-8 in transcription-independent apoptosis triggered by p53.. J Biol Chem.

[132] Moll UM, Wolff S, Speidel D, Deppert W (2005). Transcription-independent pro-apoptotic functions of p53.. Curr Opin Cell Biol.

[133] Caelles C, Helmberg A, Karin M (1994). p53-dependent apoptosis in the absence of transcriptional activation of p53-target genes.. Nature.

[134] Wagner AJ, Kokontis JM, Hay N (1994). Myc-mediated apoptosis requires wild-type p53 in a manner independent of cell cycle arrest and the ability of p53 to induce p21waf1/cip1.. Genes Dev.

[135] Lee HK, Hsu AK, Sajdak J, Qin J, Pavlidis P (2004). Coexpression analysis of human genes across many microarray data sets.. Genome Res.

[136] Bergmann S, Ihmels J, Barkai N (2004). Similarities and differences in genome-wide expression data of six organisms.. PLoS Biol.

[137] van Waveren C, Moraes CT (2008). Transcriptional co-expression and co-regulation of genes coding for components of the oxidative phosphorylation system.. BMC Genomics.

[138] di Bernardo D, Thompson MJ, Gardner TS, Chobot SE, Eastwood EL (2005). Chemogenomic profiling on a genome-wide scale using reverse-engineered gene networks.. Nat Biotechnol.

[139] Joshi A, De Smet R, Marchal K, Van de Peer Y, Michoel T (2009). Module networks revisited: computational assessment and prioritization of model predictions.. Bioinformatics.

[140] Reverter A, Hudson NJ, Nagaraj SH, Perez-Enciso M, Dalrymple BP (2010). Regulatory impact factors: unraveling the transcriptional regulation of complex traits from expression data.. Bioinformatics.

[141] Kerhornou A, Guigo R (2007). BioMoby web services to support clustering of co-regulated genes based on similarity of promoter configurations.. Bioinformatics.

[142] Draghici S, Khatri P, Tarca AL, Amin K, Done A (2007). A systems biology approach for pathway level analysis.. Genome Res.

[143] Cobb JP, Mindrinos MN, Miller-Graziano C, Calvano SE, Baker HV (2005). Application of genome-wide expression analysis to human health and disease.. Proc Natl Acad Sci U S A.

[144] Yeung KY, Medvedovic M, Bumgarner RE (2003). Clustering gene-expression data with repeated measurements.. Genome Biol.

