# Supplementary material for: Computational Identification of Transcriptional Regulators in Human Endotoxemia
Source: PLoS One. 2011 May 27;6(5):e18889. doi: 10.1371/journal.pone.0018889 (PMC3103499; doi:10.1371/journal.pone.0018889)
Supplement: Algorithms S1 — Restate the problem of discovery CRMs in gene batteries and provide detailed pseudo-code for algorithms, including the procedure ‘IsPresent’ and the algorithm of the main procedure. (DOC) [file pone.0018889.s003.doc]

**Computational identification of transcriptional regulators in human endotoxemia**

Tung T. Nguyen^1^, Panagiota T. Foteinou^2^, Steve E. Calvano^3^, Stephen F. Lowry^3^ and Ioannis P. Androulakis ^2,*^

# CRM discovery algorithms

The problem can be generally formalized as follows: given a set of N genes , each of which contains K_i_ alternative promoters whereas each promoter is represented by a list of L_ik_ binding sites (or promoter profiles) and each binding site is a 3-tuple of corresponding transcription factor name f, position p and binding orientation o , find a set of M *cis*-regulatory modules (CRMs) that are present as common over a threshold δ (70% in this study) on the set of gene promoters (M_j_ is the number of binding sites, yet to be determined, in CRM crm_j_). The statistical significance of each commonly recognized CRM vs. a background set of genes is then estimated to select only significant CRMs. The subscripts i, k, l, j indicate the gene id, the promoter id, the binding site id, and the CRM id respectively.

###### 1. The presence of a CRM on promoters of a gene – the procedure ‘IsPresent’

A small procedure that supports the main algorithm is ‘IsPresent’ which determines whether a CRM is present or not on the control region of a gene. To handle the problem that a gene may have a set of alternative promoters, we proposed a heuristic to solve the combinatorial challenge of computation. Instead of running one-by-one for each combinatorial set of promoters of N genes, procedure ‘IsPresent’ will consider K_i_ promoters of gene i at the same time and report whether the CRM is present or not on any promoter of this gene. This makes the process become feasible and produce the same results as the brute-force search for all combinations. The procedure is applicable for CRMs with a single or multiple transcription factor binding sites.

The binding site name is the corresponding name of the transcription factor family that binds to the promoter sequences. Here we made use of MatInspector to search for physical binding sites with the PWMs from MatBase version 8.0 and all optimal parameters from this database. Binding sites in a CRM are searched in order and also taken into account the binding orientation.

*1. Input: A CRM and a gene profile .*

*2. Output: - The length of the CRM if present on any promoter of gene g.*

*- If present on many promoters, only the minimum one is reported.*

*- Otherwise, return zero.*

*3. bPresent $\leftarrow$ 0*

*4. length $\leftarrow$ MAX_LENGTH*

*5. For each promoter pro_k_ of gene g*

*6. bFlag $\leftarrow$ 1*

*7. start_pos $\leftarrow$ 0*

*8. end_pos $\leftarrow$ 0*

*9. For each binding site bs_l_ in crm*

*10. If then // check the factor name and orientation*

*11. If exist > end_pos then // check the order if*

*12. end_pos*

*13. If start_pos = 0 then*

*14. start_pos*

*15. End If*

*16. End If*

*17. Else bFlag $\leftarrow$ 0*

*18. End If*

*19. Else bFlag $\leftarrow$ 0*

*20. End For*

*21. If bFlag = 1 then*

*22. bPresent $\leftarrow$ 1*

*23. length $\leftarrow$ MIN(length, end_pos – start_pos)*

*24. End If*

*25. End For*

*26. If bPresent = 1 then*

*27. Return length;*

*29. End If*

*30. Return 0;*

###### 2. Search for common CRMs – the main algorithm

Computationally, a *cis*-regulatory module (CRM) is a set of transcription factor binding sites that are characterized by their corresponding names, positions (in order and non-overlapping), and binding orientations. Instead of setting up parameters for distance variation constraints between successive binding sites, we opt to eliminate this and replace by the average length of the entire of the CRM. This parameter is calculated automatically up to the presence of this CRM on the control regions of genes in a specific gene battery. Since these CRMs need to be approximate in length, based on the average length the common level is re-calculated for those CRMs that have a length in the segment from half to double of the average length.

The algorithm has two primary steps. Step 1 will collect or detect a set of common binding sites (i.e. over a threshold δ = 70% in this case) which will be the platform for the breadth search technique in the next round. All binding sites in all promoter profiles are considered, taking into account the name and binding orientation. The binding site position is not included in specific codes of these two rounds; instead it is considered in the procedure ‘IsPresent’. Step 2 considers all possible combinations of binding sites in the resulting set of round 1. Initially, each CRM is only a binding site found in step 1. A new CRM is formed by combining the previous one and one binding site in the set of round 1. If it is still common, it will be put into the next search; otherwise it is saved to report since there is no further extension from this searching branch.

We use three sets of common CRMs to control the process. The current one contains all current CRMs that are being considered. The new one consists of all newly formed CRMs which will be considered in the next loop. And the resulting one includes all common CRMs to report. The search will be stopped whenever there is no new common CRM.

The detailed pseudo-code of the algorithm is as follows.

*1. Input: - A set of gene profiles where*

*each binding site is characterized by .*

*- A common level δ (70% in this study).*

*2. Output: - A set of common cis-regulatory modules*

***** Round 1: Select a subset of common binding sites***

*3. cBS $\leftarrow$ {} // set of common binding sites*

*4. For each bs_c_ in the set of binding sites from pro_ik_ without counting the position*

*5. Frequency $\leftarrow$ 0*

*6. For each gene g_i_ from 1 to N*

*7. If IsPresent(bs_c_, g_i_) then*

*8. Frequency $\leftarrow$ +1*

*9. End If*

*10. If Frequency/N > δ then // common TFBSs*

*11. cBS $\leftarrow$ cBS bs_c_*

*12. End IF*

*13. End For*

****** Round 2: Finding all possible common CRMs***

*14. res_cCRM $\leftarrow$ {} // the resulting set of common cis-regulatory modules*

*15. cur_cCRM $\leftarrow$ cBS // the current set of common cis-regulatory modules*

*16. While cur_cCRM <> {} do*

*17. new_cCRM $\leftarrow$ {} // the new set of common CRMs for the next loop*

*18. For each CRM crm_j_ in cur_cCRM*

*19. For each common binding site bs_c_ in cBS*

*20. new_CRM $\leftarrow$ crm_j_ bs_c_ // extend the CRM*

*21. Frequency $\leftarrow$ 0*

*22. Length_set = {}*

*23. For each gene g_i_ from 1 to N*

*24. length $\leftarrow$ IsPresent(new_CRM, g_i_)*

*25. If length >0 then*

*26. Frequency $\leftarrow$ +1*

*27. total_length $\leftarrow$ +length*

*28. Length_set $\leftarrow$ Length_set {length}*

*29. End If*

*30. End For*

*31. average_length $\leftarrow$ total_length/Frequency*

*32. Frequency $\leftarrow$ 0 // recalculating the common level*

*33. For each length L_p_ in the Length_set*

*34. If average_length < 2*L_p_ and L_p_ < 2*average_length then*

*35. Frequency $\leftarrow$ +1*

*36. End If*

*37. End For*

*38. If Frequency > δ then*

*39. new_cCRM $\leftarrow$ new_cCRM {new_crm} // add the new one*

*40. End If*

*41. Else*

*42. res_cCRM $\leftarrow$ res_cCRM {crm_j_} // save to report, no further search*

*43. End Else*

*44. End For*

*45. End For*

*46. cur_cCRM $\leftarrow$ new_cCRM // reset the set of common CRMs for the next loop*

*47. End While*

*48. Return res_cCRM;*
