# Supplementary material for: Computational Identification of Transcriptional Regulators in Human Endotoxemia
Source: PLoS One. 2011 May 27;6(5):e18889. doi: 10.1371/journal.pone.0018889 (PMC3103499; doi:10.1371/journal.pone.0018889)
Supplement: Appendix S1 — Provide an illustration to prove that the set of common CRMs found by the proposed heuristic is the same with the set of common CRMs found from all promoter combinations. (DOC) [file pone.0018889.s004.doc]

**Computational identification of transcriptional regulators in human endotoxemia**

Tung T. Nguyen^1^, Panagiota T. Foteinou^2^, Steve E. Calvano^3^, Stephen F. Lowry^3^ and Ioannis P. Androulakis ^2,*^

# Appendix – Promoter combination problem

**Lemma 1:** Given that each promoter combination in a set of N genes is defined as , let $C^{N}$ be the set of all promoter combinations , and $S_{0}$be the union set of common CRMs found from all promoter combinations in $C^{N}$. Prove that $S\equiv S_{0}$ where $S$ is the set of common CRMs found from the combination . Since a CRM is a set of binding sites present on an individual promoter, operator $, \vee$are only applied at the level of promoter i.e. elements of promoter profiles are not affected. Therefore, instead of proving $S\equiv S_{0}$ we will prove that .

***Proof:***

1. For N = 2:

The illustration can be described shortly as follows

Therefore, it is true that the set of common CRMs found in the combination $F^{2}$ is also the set of common CRMs found from all promoter combinations of these two genes.

1. For N = 3:
2. Assume that the lemma is true with N = n-1, we now prove it is true with N = n.
