# Supplementary material for: Computational Identification of Transcriptional Regulators in Human Endotoxemia
Source: PLoS One. 2011 May 27;6(5):e18889. doi: 10.1371/journal.pone.0018889 (PMC3103499; doi:10.1371/journal.pone.0018889)
Supplement: Procedures S1 — Provide source codes of some main procedure (by Perl language) including (1) check the existence of a CRM on alternative promoters of a gene, (2) search for common CRMs in a gene battery, and (3) estimate the hyper-geometric p-value of CRMs vs. the background set. (DOC) [file pone.0018889.s005.doc]

**Computational identification of transcriptional regulators in human endotoxemia**

Tung T. Nguyen^1^, Panagiota T. Foteinou^2^, Steve E. Calvano^3^, Stephen F. Lowry^3^ and Ioannis P. Androulakis ^2,*^

# Source code of main procedures (Perl language)

1. ***Check the existence of a cis-regulatory module on alternative promoters of a gene***

# Check to know whether this gene contains CRM A

# Input: gene name and the CRM under a string format

# Output: the length of the CRM on this gene if existed; 0 otherwise

# %pro2idx and @pro_profiles are two external variables that indicate the index of the

# corresponding promoter profiles and the promoter profiles

sub IsPresent{

my ($gene, $CRM) = @_;

my (%profile, @TFs, @fields, @zfields, $key);

my ($len, $bFlag, $yes, $idx, $i, $j, $z);

my (@pos1, @pos2, $pos, $agree, $k, $l);

# obtain the list of participated TFBSs with corresponding binding orientation

@TFs = split(/__/, $CRM);

# check to see whether this CRM appears at least in one alternative promoter

# of the gene or not

$bFlag = 0; $len = 0;

@fields = split(/,/, $gene2pro{$gene}); # obtain all alternative promoters

for ($i=0;$i<=$#fields;$i++){

# construct the profile for the current promoter

$idx = $pro2idx{$fields[$i]};

%profile = ();

@zfields = split(/,/, $pro_profiles[$idx]);

for ($z=0;$z<=$#zfields;$z+=2){ $profile{$zfields[$z]}=$zfields[$z+1]; }

# check whether all TFBSs in this module exist on this promoter

$yes = 1; $l = 0;

foreach $key (@TFs){

if (exists($profile{$key})){ $l = 1; }

else{ $yes = 0; }

}

if ($yes==1 and $#TFs>0){

$pos = -1;

# check the order of TFBSs

for ($j=0;$j<$#TFs;$j++){

@pos1 = split(/__/, $profile{$TFs[$j]});

@pos2 = split(/__/, $profile{$TFs[$j+1]});

if ($pos==-1){ $pos = $pos1[0]; }

$agree = -1;

for ($k=0;$k<=$#pos2;$k++){

if ($pos<$pos2[$k]){

if ($agree==-1){ $agree = $k; }

}

}

if ($agree==-1){ $yes = 0; }

else{

# take the largest position on the left side of the module

if ($j==0){

for ($k=0;$k<=$#pos1;$k++){

if ($pos1[$k]<$pos2[$agree]){ $pos = $pos1[$k]; }

}

$l = $pos;

}

if ($j==($#TFs-1)){ $l = $pos2[$agree] - $l; }

$pos = $pos2[$agree];

}

}

}

if ($yes==1){ $bFlag = 1;

if ($len==0){ $len = $l; } else{ $len = MIN($len,$l); }

}

}

return $len;

}

1. ***Search for common cis-regulatory modules in a gene battery***

# Search for all putative CRMs present on promoters of a group of genes

# Input: the gene group

# Output: the list of common CRMs %comCRMs and corresponding frequencies %CRMFreq

# $fComLevel: 70% - the common level

sub search_comCRMs{

my $groupid = uc($_[0]);

my (@genes, %CRMs, @comTFs, $N, $common);

my (%CRMs, %comCRMs, @curCRMs, $newCRM, $bFlag);

my (%profile, @fields, $idx, $str, $key);

my (@len, $avglen, $freq, $i, $j, $k, $l);

# take a list of genes

$str = $group2gene{$groupid};

@genes = split(/,/, $str);

$N = $#genes + 1; if ($N<=0){ return; }

$common = $fComLevel*$N;

%comCRMs = (); %CRMFreq = (); %CRMs = ();

# obtain the list of all participated TFBSs

for ($i=0;$i<$N;$i++){

@fields = split(/,/, $gene2pro{$genes[$i]}); # obtain all alternative promoters

for ($j=0;$j<=$#fields;$j++){

$idx = $pro2idx{$fields[$j]};

# construct the profile for the current promoter

%profile = ();

my @zfields = split(/,/, $pro_profiles[$idx]);

for (my $z=0;$z<=$#zfields;$z+=2){ $profile{$zfields[$z]}=$zfields[$z+1]; }

# accumulate the list of current TFBSs

foreach $key (keys %profile){ $comCRMs{$key} = 1; }

}

}

print "Common TFBSs:\n";

# initialize the list of single common TFBSs

@curCRMs = (keys %comCRMs); %comCRMs = ();

foreach $key (@curCRMs){

$freq = 0;

for ($i=0;$i<$N;$i++){

if (IsPresent($genes[$i], $key)){ $freq++; }

}

if ($freq>=$common){

$comCRMs{$key} = $freq/$N;

$CRMs{$key} = 1;

print "$key\n";

}

}

%CRMFreq = %comCRMs;

if ($N<=2){ return; } # $N - the number of genes in this gene battery

print "New common cis-regulatory modules:\n";

# run a routine, stop if there is no new CRM formed

$bFlag = 1; @comTFs = (keys %comCRMs);

while ($bFlag==1){

$bFlag = 0;

# find comCRMs in curCRMs

@curCRMs = (keys %comCRMs); %comCRMs = ();

print "\n\tNumber of current CRMs: $#curCRMs+1\n";

for ($i=0;$i<=$#curCRMs;$i++){

for ($j=0;$j<=$#comTFs;$j++){

# create new CRMs by combining two old CRMs

$newCRM = combineCRM($curCRMs[$i], $comTFs[$j]); # ORDER

if ($CRMs{$newCRM} eq ""){

# check if common --> save

$freq = 0; @len = (); $avglen = 0;

for ($k=0;$k<$N;$k++){

$l = IsPresent($genes[$k], $newCRM);

if ($l>0){ push @len, $l; }

}

# estimate the CRM average length

$avglen = 0;

for ($k=0;$k<=$#len;$k++){ $avglen += $len[$k]; }

if ($avglen>0){ $avglen /= ($#len+1); }

# count the frequency of comCRMs between avglen/2 to 2*avglen

for ($k=0;$k<=$#len;$k++){

if ($avglen/2<$len[$k] and $len[$k]<2*$avglen){

$freq++;

}

}

if ($freq>=$common){

$bFlag = 1; $comCRMs{$newCRM} = 1;

$CRMs{$newCRM} = 1;

# insert the new one and delete the old one

$CRMFreq{$newCRM} = $freq/$N;

$CRModules{$newCRM} = "$avglen\__$min_len\__$max_len";

delete($CRMFreq{$curCRMs[$i]});

delete($CRMFreq{$comTFs[$j]});

delete($CRModules{$curCRMs[$i]});

delete($CRModules{$comTFs[$j]});

# keep only the largest one

# delete all CRMS that are part of the right-side of this CRM

@fields = split(/__/, $newCRM);

for ($k=1;$k<$#fields;$k++){

$str = join("__", @fields[$k..$#fields]);

delete($CRMFreq{$str});

delete($CRModules{$str});

}

}

else{ $CRMs{$newCRM} = 0; }

}

}

}

}

}

# combine two cis-regulatory modules into one new

sub combineCRM{

my ($CRM1, $CRM2) = @_;

my (@fields, $newCRM);

$newCRM = "$CRM1\__$CRM2";

# sort to avoid redundancy in order

@fields = split(/__/, $newCRM);

@fields = sort(@fields);

$newCRM = $fields[0];

for (my $i=1;$i<=$#fields;$i++){

$newCRM = "$newCRM\__$fields[$i]";

}

return $newCRM;

}

1. ***Estimate the hyper-geometric p-value of CRMs vs. the background set***

# Estimate the hyper-geometric p-value from the background set (5000 genes)

# $n is the number of genes in the gene battery; $N is the number of background genes

# %comCRMs is an external hash which consists of all recognized CRMs (keys) and

# corresponding min and max length (values) in this gene battery

# %CRMFreq is an external hash which consists of all recognized CRMs (keys) and

# corresponding frequencies (values) in this gene battery

sub count_in_background{

my ($n, $N) = @_;

my (%pvalCRMs, @CRMs, $N, $pvalue, $i, $k);

my (@tmp, $len, $min_len, $max_len, $freq);

@CRMs = keys %comCRMs; # extract all recognized CRMs

# count the existence of each CRM in this set of genes

for ($i=0;$i<=$#CRMs;$i++){

@tmp = split(/__/, $comCRMs{$CRMs[$i]});

# estimate the consistent length of a CRM on background genes

# with $len_percent = 10%

$min_len = $tmp[1] - $tmp[1]/$len_percent;

$max_len = $tmp[2] + $tmp[2]/$len_percent;

$freq = 0;

for ($k=0;$k<$N;$k++){

$len = IsPresent($bkgnd_genes[$k], $CRMs[$i]);

if ($min_len<$len and $len<$max_len){ $freq++; }

}

$k = int($n*$CRMFreq{$CRMs[$i]}+0.5);

$pvalue = hyperCumulative($k, $n, $freq, $N);

$pvalCRMs{$CRMs[$i]} = $pvalue;

}

return (%pvalCRMs);

}

# Estimate the hyper-geometric probability of obtaining

# $nc genes regulated by cis-regulatory module A in $Nc coexpressed genes

# in a population of $N genes with $n genes regulated by A

sub hypergeometric{

my ($i, $Nc, $n, $N) = @_;

my $nr = $N - $n;

return 0 unless ($n>0 and $n<=$N and $Nc>0 and $Nc<=$N);

return 0 unless $i<=$Nc and $i<=$n;

return choose($n, $i) * choose($nr, $Nc - $i) / choose($nr + $n, $Nc);

}

# Binomial coefficient

sub choose{

my ($n, $k) = @_;

my ($res, $j) = (1, 1);

return 0 if $k > $n || $k < 0;

$k = ($n - $k) if ($n - $k) < $k;

while ($j <= $k){

$res *= $n--;

$res /= $j++;

}

return $res;

}

# Calculate the cumulative hyper-geometric probability

sub hyperCumulative{

my ($nc, $Nc, $n, $N) = @_;

my $p_value = 0;

my $nm = $n; $nm = $Nc if ($Nc<$n);

for(my $i=$nc; $i<=$nm; $i++){

$p_value += hypergeometric($i, $Nc, $n, $N);

}

return $p_value;

}
